# Identification and characterization of wheat stem rust resistance gene *Sr21* effective against the Ug99 race group at high temperature

**DOI:** 10.1371/journal.pgen.1007287

**Published:** 2018-04-03

**Authors:** Shisheng Chen, Wenjun Zhang, Stephen Bolus, Matthew N. Rouse, Jorge Dubcovsky

**Affiliations:** 1 Department of Plant Sciences, University of California, Davis, Davis, CA, United States of America; 2 USDA-ARS Cereal Disease Laboratory and Department of Plant Pathology, University of Minnesota, St. Paul, MN, United States of America; 3 Howard Hughes Medical Institute, Chevy Chase, MD, United States of America; University of Zurich, SWITZERLAND

## Abstract

Wheat stem rust, caused by *Puccinia graminis* f. sp. *tritici* (*Pgt*), is a devastating foliar disease. The Ug99 race group has combined virulence to most stem rust (*Sr*) resistance genes deployed in wheat and is a threat to global wheat production. Here we identified a coiled-coil, nucleotide-binding leucine-rich repeat protein (NLR) completely linked to the Ug99 resistance gene *Sr21* from *Triticum monococcum*. Loss-of-function mutations and transgenic complementation confirmed that this gene is *Sr21*. *Sr21* transcripts were significantly higher at high temperatures, and this was associated with significant upregulation of pathogenesis related (*PR*) genes and increased levels of resistance at those temperatures. Introgression of *Sr21* into hexaploid wheat resulted in lower levels of resistance than in diploid wheat, but transgenic hexaploid wheat lines with high levels of *Sr21* expression showed high levels of resistance. *Sr21* can be a valuable component of transgenic cassettes or gene pyramids combining multiple resistance genes against Ug99.

## Author summary

Wheat stem rust is a devastating disease that is threatening global wheat production. The emergence of new virulent races of this pathogen in Africa, including the Ug99 race group, has prompted global efforts to find effective resistance genes. We report here the identification of stem rust resistance gene *Sr21* that is effective against the Ug99 race group. We developed a diagnostic marker to accelerate its deployment in wheat breeding programs and demonstrated that the introduction of two *Sr21* copies in transgenic wheat results in high levels of resistance. An unusual characteristic of *Sr21* is its increased resistance to stem rust at high temperatures. We show here that this is associated with the ability of *Sr21* to coordinate the upregulation of multiple pathogenesis related genes at high temperatures. These genes slow down the growth of the pathogen and result in the characteristic *Sr21* intermediate resistance reaction at high temperatures. A better understanding of this temperature dependent resistance mechanism will be useful for controlling the rust pathogens in our changing environments.

## Introduction

More than 700 million tons of wheat are produced every year, but further increases are needed to feed a rapidly growing human population. Reducing yield losses caused by wheat pathogens can contribute to this goal. Wheat stem rust, caused by the fungal pathogen *Puccinia graminis* f. sp. *tritici* (henceforth *Pgt*), poses an urgent threat to global wheat production since new virulent races have overcome widely deployed resistance genes.

In 1998, a new *Pgt* race was detected in Uganda that was virulent to the widely deployed stem rust resistance genes *Sr31* and *Sr38* [1]. This race, also known as TTKSK using the North American system of nomenclature for *Pgt* races [2,3], was virulent to roughly 90% of the global wheat cultivars [4]. Since then, the Ug99 race group has spread to more than 13 countries in Africa and the Middle East [4–8] and acquired virulence to additional resistance genes (*Sr24*, *Sr36*, *Sr9h* and *SrTmp* [3,9–11]), prompting efforts to identify and clone effective resistance genes.

The large and complex nature of the wheat genome has limited the number of cloned Ug99 resistance genes to six, including *Sr35* [12], *Sr33* [13], *Sr50* [14], *Sr22* [15], *Sr45* [15], and *Sr13* [16]. More resistance genes are necessary to diversify the combinations of *Pgt* resistance genes deployed as gene pyramids or in transgenic cassettes to provide durable resistance [15,17,18].

The stem rust resistance gene *Sr21* was discovered in diploid wheat *Triticum monococcum* (genome A^m^), which is closely related to *T*. *urartu*, the progenitor of the A genome in polyploid wheat [19]. *Sr21*, which confers a more effective resistance to the Ug99 race group at high temperatures (20–24°C) than at low temperature (16°C) [20], was transferred to hexaploid wheat (*T*. *aestivum*) [21]. We previously mapped *Sr21 *to a 0.19 cM interval on the central region of chromosome arm 2A^m^L and showed that this region includes a cluster of NBS-LRR resistance genes in the colinear region in the A genome of *T*. *aestivum* [20].

Here, we report the identification, validation and characterization of the wheat stem rust resistant gene *Sr21*, which encodes a coiled-coil nucleotide-binding leucine-rich repeat protein (NLR). Six pathogenesis-related (*PR*) genes showed increased transcript levels in *Sr21-*resistant genotypes inoculated with *Pgt*, only when plants were grown at high temperatures. This result provides a tentative explanation for the more effective *Pgt* resistance conferred by *Sr21* at high temperatures. Finally, we identified five different resistance haplotypes of *Sr21* and developed a diagnostic molecular marker to accelerate its deployment in breeding programs.

## Results

### High-density genetic and physical maps of the *Sr21* region

**Fig 1:**
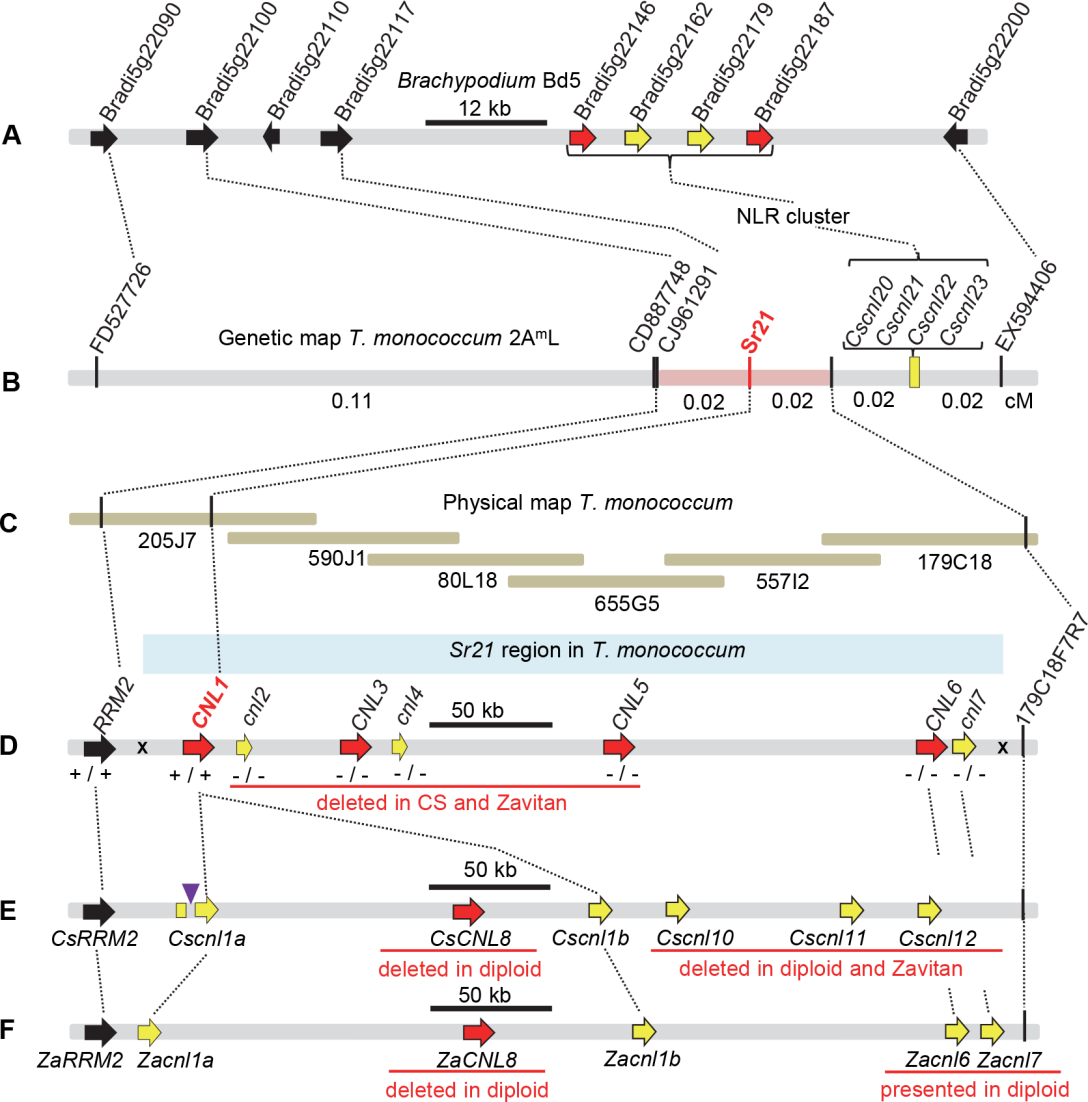
Map-based cloning of *Sr21*. (A) *Brachypodium distachyon* chromosome 5 region colinear with the *Sr21-*candidate region. Arrows in bracket indicate NLR genes and pseudogenes. **(B)** High-density genetic map of *Sr21* on chromosome arm 2A^m^L. **(C)** Physical map of the *Sr21* region constructed with overlapping BACs from diploid wheat accession DV92. **(D)** Diagrammatic representation of the annotated sequence of the *Sr21* region (GenBank accession MG582649). NLR genes are represented by red arrows and capital letters (*CNL*) and pseudogenes by yellow arrows and lower-case letters (*cnl*). Similar NLR numbers indicate putative orthologous genes. “+” and “-” signs below gene names indicate detection of expression in resistant *T*. *monococcum* accessions DV92 (first sign) and G3116 (second sign). **(E)** Genes in the colinear region of *T*. *aestivum* cv. Chinese Spring. **(F)** Genes in the colinear region of *T*. *turgidum* ssp. *dicoccoides* Zavitan [23]. NLRs with the same number are putative orthologs.

The 0.19 cM region on *T*. *monococcum* chromosome arm 2A^m^L including *Sr21* is colinear with an 88-kb region in *Brachypodium distachyon* chromosome 5 flanked by genes *Bradi5g22090* and *Bradi5g22200* [20]. In the present study, we identified the wheat orthologs of six *B*. *distachyon* genes present in this colinear region (*Bradi5g22100*, *Bradi5g22117*, *Bradi5g22146*, *Bradi5g22162*, *Bradi5g22179* and *Bradi5g22187*, Fig 1A, S1 Table) and mapped them in ten wheat lines with recombination events between *Sr21* flanking markers *FD527726* and *EX594406* (Fig 1B). Using these new markers, *Sr21* was mapped 0.02 cM distal to *CJ961291* and 0.04 cM proximal to a group of four linked NLR pseudogenes (*Cscnl20*, *Cscnl21*, *Cscnl22* and *Cscnl23*, Fig 1B).

We screened a bacterial artificial chromosome (BAC) library of the resistant *T*. *monococcum* accession DV92 [22] using the closest markers, but sequencing of selected BACs showed no connection between proximal and distal groups (S1 Fig). We initiated a chromosome walk from the closest marker (*CJ961291*, 0.02 cM from the phenotype) in the proximal group (Fig 1C, BAC 205J7). Marker *CJ961291* (*RRM2*) showed a recombination event with gene *CNL1*, which was completely linked to the phenotype, completing the proximal side of the physical map. After five cycles of library screening, BAC sequencing and marker development, we identified an additional recombination event between the phenotype and marker *179C18F7R7*, which closed the distal end of the physical map.

Sequencing the 405-kb candidate region (Fig 1D, blue area, S1 Fig red colored BACs, GenBank accession MG582649) revealed four complete NLR genes (henceforth, *CNL1*, *CNL3*, *CNL5*, and *CNL6*) and three NLR truncated genes with premature stop codons (henceforth, *cnl2*, *cnl4*, and *cnl7*).

Multiple rearrangements were observed in this NLR cluster in *T*. *aestivum* cv. Chinese Spring (419 kb, Fig 1E, IWGSC RefSeq v1.0) and *T*. *turgidum* ssp. *dicoccoides *Zavitan (435 kb, Fig 1F, Zavitan WEWSeq v1.0) [23]. The two polyploid species included the complete gene *CsCNL8* (*ZaCNL8*) that was absent in *T*. *monococcum*, whereas the *T*. *monococcum* region including *cnl2*, *CNL3*, *cnl4* and *CNL5* was missing in Chinese Spring and Zavitan (Fig 1E and 1F). NLR pseudogenes *Cscnl10*, *Cscnl11* and *Cscnl12* identified in CS were not detected in *T*. *monococcum* or Zavitan. The CS and Zavitan pseudogenes *Cscnl1a* (*Zacnl1a*) and *Cscnl1b* (*Zacnl1b*) were similar to *T*. *monococcum CNL1* (~99% identity). *Cscnl1a* was interrupted by the insertion of a repetitive sequence in CS but not in Zavitan (Fig 1E and 1F).

### Natural variation in NLR genes linked to *Sr21*

We sequenced the four NLR genes completely linked to the *Sr21* phenotype (*CNL1*, *CNL3*, *CNL5* and *CNL6*) from diploid accessions G3116 (*Sr21*, resistant) and PI 272557 (no-*Sr21*, susceptible). Using seven different pairs of gene-specific primers, we amplified *CNL3* and *CNL5* from DV92 but not from G3116 and PI 272557 (similar to CS and Zavitan, Fig 1E and 1F). Since G3116 carries the *Sr21* resistant allele, the absence of these two genes suggested that *CNL3* and *CNL5 *were unlikely candidate genes for *Sr21*. *CNL6* was PCR-amplified in all three accessions, but G3116 showed a premature stop codon at position 742 (Q742*), suggesting that this gene was not *Sr21*. By contrast, *CNL1* was PCR-amplified from both resistant accessions (DV92 and G3116) but not from *Pgt* susceptible accession PI 272557.

Taken together, these results suggested that *CNL1* was the best candidate gene for *Sr21* among the NLR genes linked to *Sr21*. This hypothesis was further supported by expression data for these genes in transcriptome databases of DV92 and G3116 [24]. Among the four NLR genes completely linked to *Sr21*, we only detected transcripts for *CNL1* in both DV92 and G3116 databases (Fig 1D).

### Validation of *CNL1* using EMS mutants

**Fig 2:**
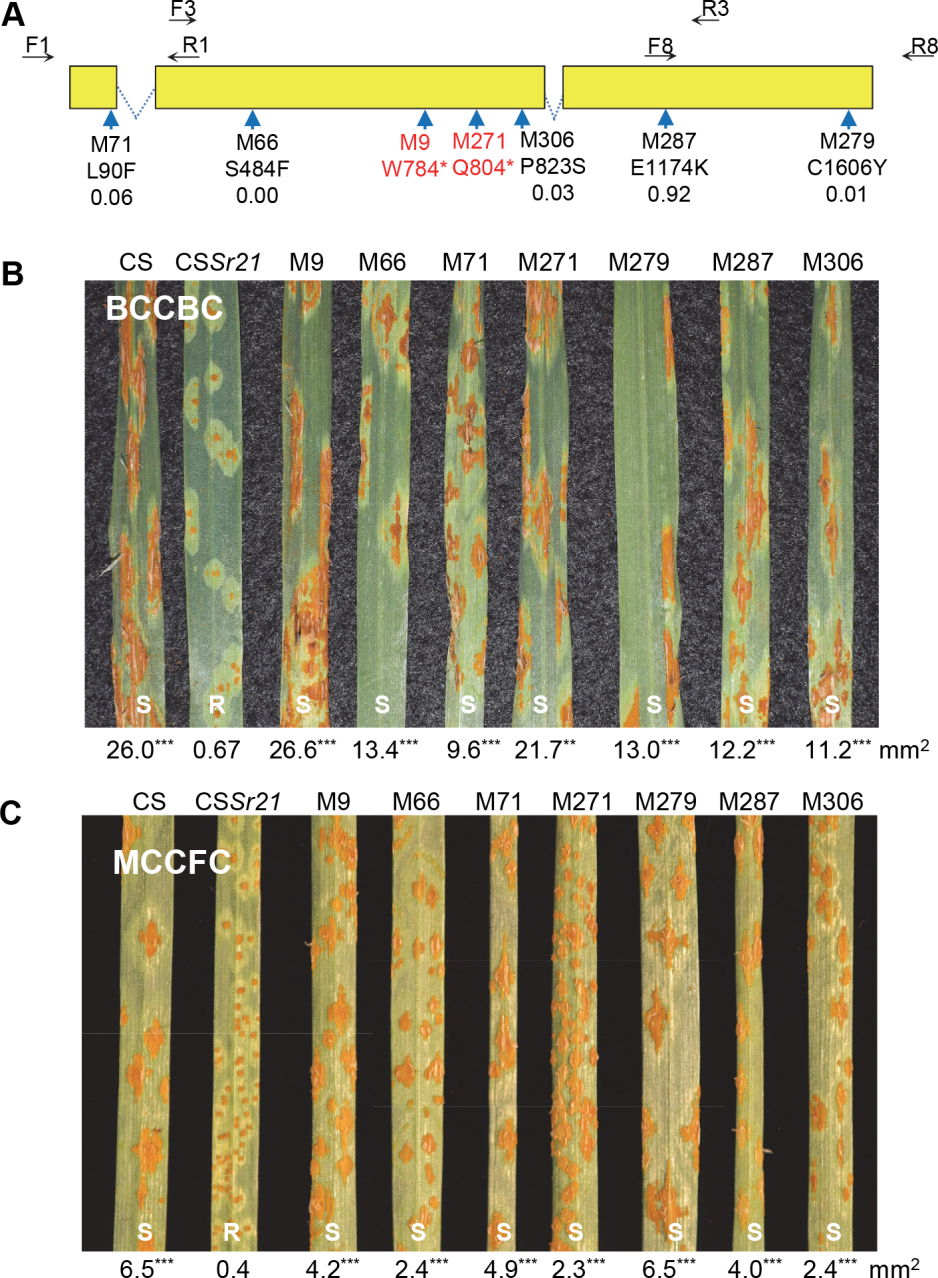
Validation of the *Sr21* candidate gene using EMS mutants. **(A)** Structure of gene *CNL1* (from start codon to stop codon). Primers used for haplotyping are indicated by black arrows. Dotted lines indicate introns, and yellow rectangles indicate coding exons. The positions of the mutations are indicated by blue arrows. Mutation names in red indicate premature stop codons and in black indicate amino acid changes, which are indicated below the names with the corresponding SIFT scores “Sorting intolerant from tolerant” scores lower than 0.05 are predictive of deleterious missense mutations [50]). **(B)** Infection types on Chinese Spring (CS), CS*Sr21*, and seven mutants inoculated with *Pgt* race BCCBC. Numbers below leaves indicate average pustule sizes (n = 3) and superscripts indicate significance of differences with CS*Sr21* (*** = *P*< 0.001). **(C)** Same as B above but with race MCCFC.

To test if *CNL1* was required for *Sr21* resistance, we mutagenized the *Sr21* introgression lines in Chinese Spring (henceforth CS*Sr21*) with ethyl methane sulfonate (EMS) and generated 1,151 M_1_ mutant plants. Among the 1,151 M_2_ mutant families screened with race BCCBC, we identified seven showing susceptible plants (M9, M66, M71, M271, M279, M287 and M306, Fig 2A and 2B). M_3_ seeds from the susceptible plants were also susceptible to *Pgt* race MCCFC (Fig 2C).

Sequencing of *CNL1* from the seven susceptible mutants (primers in S1 Table) revealed amino acid changes in five mutants and premature stop codons in two mutants (Fig 2A). Since the probability of a truncation or missense mutation in an annotated gene in a hexaploid wheat line mutagenized with 0.8% EMS is roughly 0.02 [25], the probability of detecting such changes in seven independent mutants by chance is < 1.3×10^−12^. These results demonstrated that *CNL1* is required for *Sr21* resistance to *Pgt*.

### Wheat plants transformed with *CNL1* were resistant to Ug99

**Fig 3:**
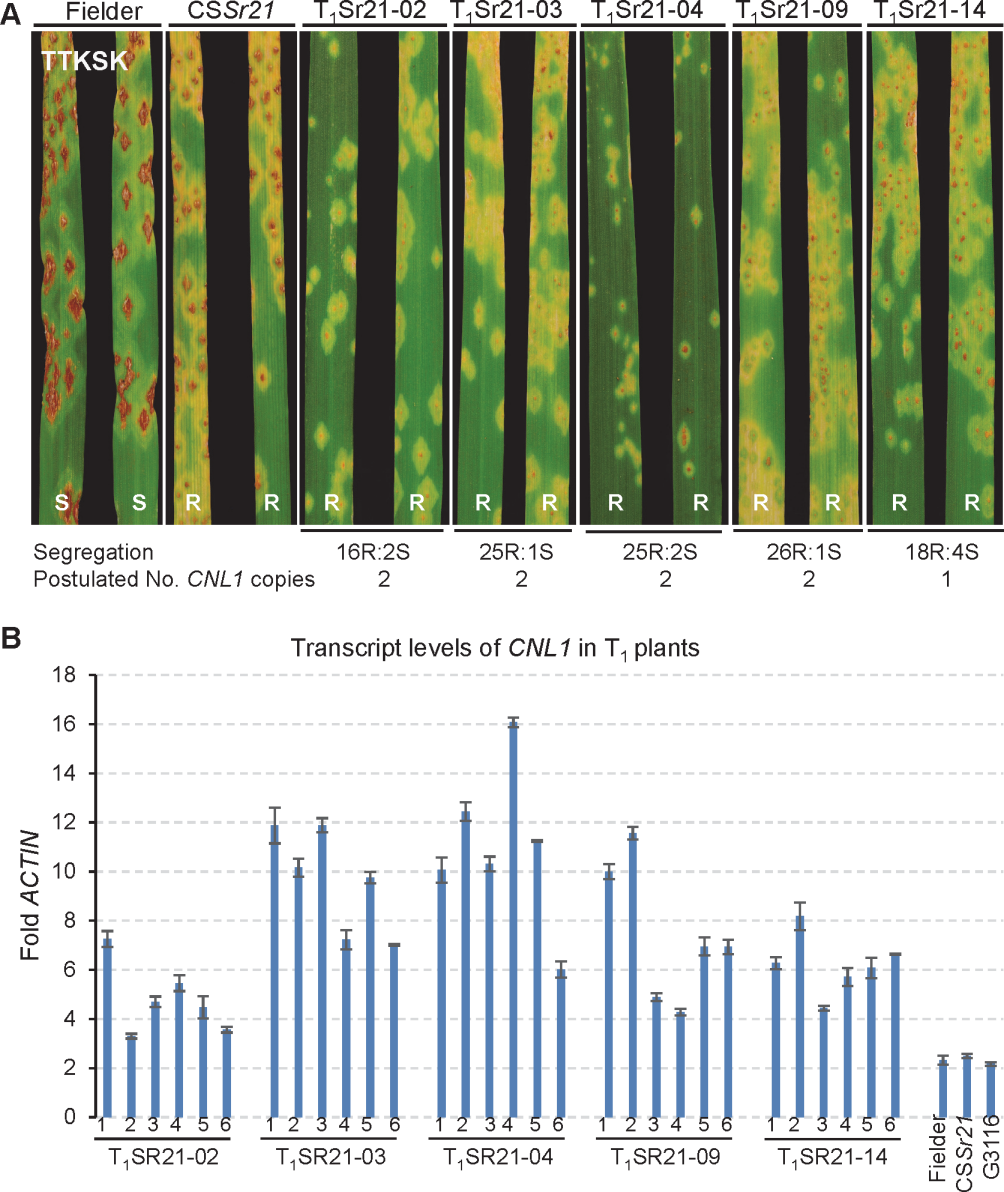
*Sr21* transgenic plants. **(A)** Reactions to *Pgt* race TTKSK (Ug99) in Fielder, CS*Sr21*, and transgenic families T_1_Sr21-02, T_1_Sr21-03, T_1_Sr21-04, T_1_Sr21-09 and T_1_Sr21-14. S = Susceptible, R = Resistant. Plants were grown at 25°C during the day and 22°C during the night. The numbers below the figure indicate the observed segregation of resistant or partial resistant versus susceptible lines and the postulated number of independent genes based on χ^2^ tests (S2 Table). **(B)** Transcript levels of *CNL1* in transgenic T_1_ families T_1_SR21-02, T_1_SR21-03, T_1_SR21-04, T_1_SR21-09 and T_1_SR21-14 (six plants per event). Transcript levels are expressed as fold-*ACTIN* using the 2^-ΔCT^ method. Fielder has the susceptible *CNL1* allele S2, which is expressed at similar levels as the resistant allele in hexaploid CS*Sr21* and diploid *T*. *monococcum* G3116.

To test if *CNL1* was also sufficient to confer resistance to *Pgt*, we generated thirteen independent transgenic events (T_0_Sr21) in the susceptible common wheat variety Fielder. Among the 13 T_0_ plants, we prioritized five that showed higher transcript levels of *CNL1* than Fielder, which has a non-functional copy of *CNL1* (S2 Fig). The T_1_ progenies from these five events showed segregation for resistance when challenged with *Pgt* race TTKSK (isolate 04KEN156/04). Some of the resistant transgenic plants showed even better levels of resistance than the CS*Sr21* positive control, which carries a *Sr21* introgression from *T*. *monococcum* (Fig 3A). These results confirmed that *CNL1* is sufficient to confer resistance to TTKSK. Taken together, the high-density map, the mutants and the transgenic results confirmed that *CNL1* is *Sr21*.

### *CNL1* copy number and expression in transgenic plants

The observed segregation of resistant and susceptible T_1_ transgenic plants (Fig 3A) suggested that four of these five transgenic lines have more than one independent functional *CNL1* insertions. Genotypes from roughly 50 T_1_ plants from each event showed significant departures from the expected 3:1 segregation ratio (*P* < 0.001, S2 Table), which suggested the presence of three to six copies of *CNL1*. This was validated by TaqMan copy number assays [26] (S2 Table). The larger copy number estimates obtained from the genotypic data than from the phenotypic data is likely explained by the presence of non-functional *CNL1* insertions and/or by the presence of linked functional copies.

On average, the five selected T_1_ transgenic events showed 2.6- to 5.7-fold higher *CNL1* transcript levels than CS*Sr21* (S2 Table and Fig 3B), confirming the T_0_ results (S2 Fig). Copy number based on the *CNL1* TaqMan assay was significantly correlated with average transcript levels in the five selected transgenics and CS*Sr21* (*R* = 0.83, *P* = 0.039). A similar correlation (*R* = 0.81, *P* = 0.0009) was observed between the T_0_ expression levels from all 13 transgenic lines and the copy number estimated by the TaqMan assay (S2 Fig). These results suggest that the differences in expression are driven, at least in part, by differences in *CNL1* copy number. This was also reflected in the levels of resistance to TTKSK, where a negative correlation was observed between *CNL1* copy number and average rust pustule size (*R* = -0.92, *P* < 0.0001, S3 Fig). In this experiment, we also compared the *CNL1* transcript levels between the *Sr21-*resistant accessions G3116 (diploid) and CS*Sr21* (hexaploid) and found no significant differences (Fig 3B).

### *CNL1* structure and alternative splicing forms

We compared the sequences of the *CNL1* transcripts (from the DV92 and G3116 transcriptome database [24]) with the corresponding genomic sequences and determined that the 4,872 bp coding sequence of *CNL1* is divided in three exons that encode 1,624 amino acids. The predicted protein includes an N-terminal coiled-coil (CC) domain, a central nucleotide-binding (NB) site, and a leucine-rich repeat (LRR) region, typical of many NLR proteins. A nuclear localization signal (NLS) was predicted in Sr21 between amino acids 141 and 152 (cNLS Mapper http://nls-mapper.iab.keio.ac.jp/cgi-bin/NLS_Mapper_form.cgi).

Comparing different transcripts and genomic sequences, we identified a 210-bp 5’ untranslated region (UTR) and a 2,159-bp 3’ UTR. The 5’ UTR does not include any introns, whereas the 3’ UTR shows 2 to 4 introns depending on the alternative splice forms (S4 Fig). These results were confirmed by 5’ and 3’ rapid amplification of cDNA ends (5’ and 3’ RACE). We identified 10 *CNL1* alternative splicing forms, which differ only in their 3’ UTR regions (S4 Fig). We detected five alternative splicing forms (*CNL1-*1 to *CNL1-*5) in the transcriptomes of DV92 and G3116 [24] and an additional five (*CNL1-*6 to *CNL1-*10) in the 3' RACE reactions.

Since *Sr21* resistance is modulated by temperature [20], we characterized the frequency of the different alternative splice forms in 3' RACE reactions from RNAs obtained from G3116 *T*. *monococcum* resistant plants grown at 24°C and 16°C. The plants at each temperature were further divided in mock-inoculated and inoculated with *Pgt* race BCCBC, and RNA samples were extracted six days after inoculation (80 clones per treatment were sequenced). χ^2^ tests showed no significant differences (*P* = 0.81) in the frequencies of alternative splice forms in inoculated *vs*. mock-inoculated plants (averaged across temperatures) but detected significant differences between temperatures (*P* = 0.002, averaged across inoculation treatments, S3 Table). The *CNL1-*1, *CNL1-*5 and *CNL1-*6 forms were more frequent at 24°C than at 16°C, and the opposite was observed for *CNL1-*2. We currently do not know the biological significance of these differences.

### Effect of temperature and *Pgt* inoculation on transcript levels of *CNL1* and *PR* genes

**Fig 4:**
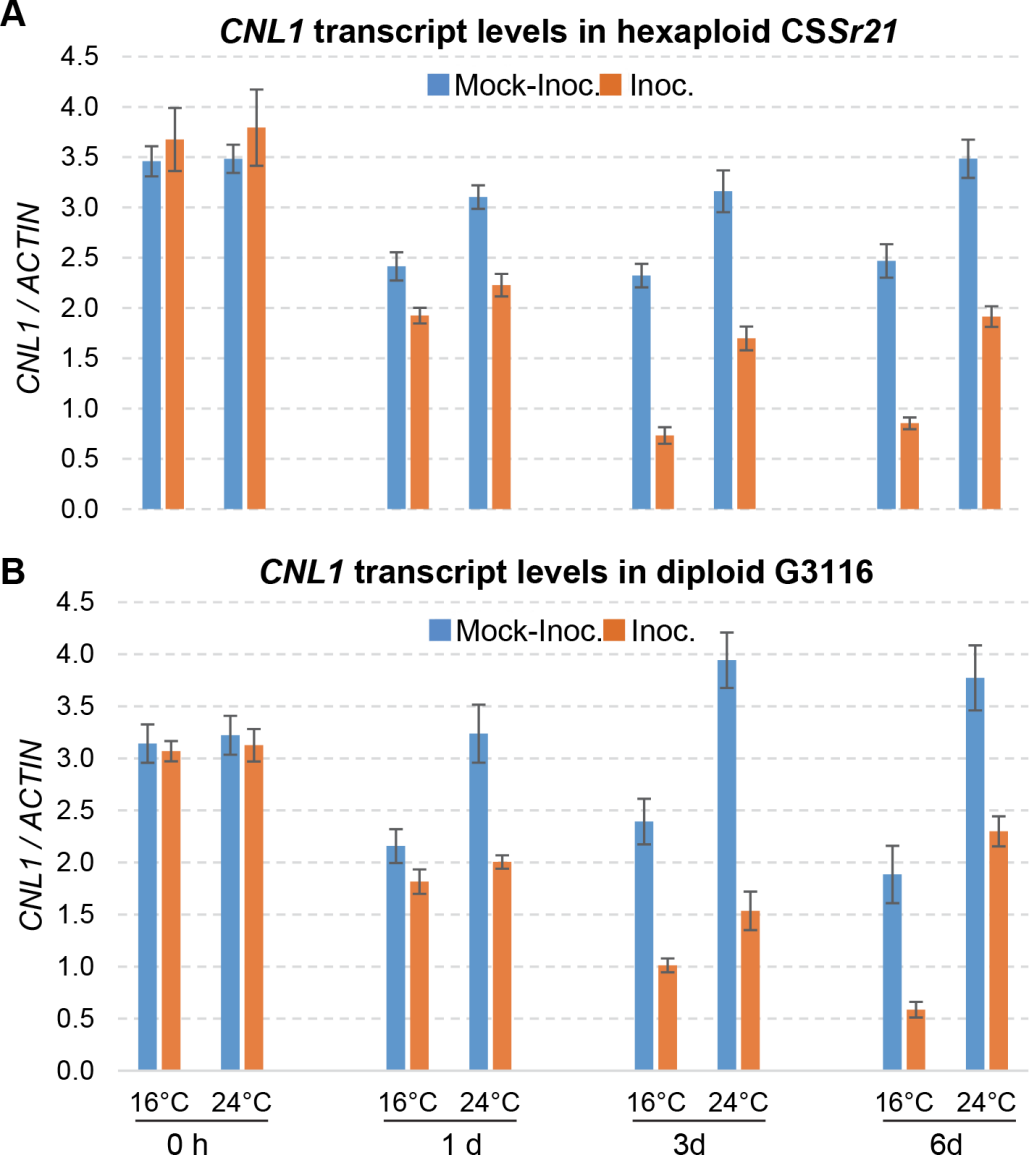
Effect of temperature and *Pgt* inoculation (race BCCBC) on *CNL1* transcript levels. **(A)** *Sr21* resistant hexaploid wheat CS*Sr21*. **(B)** *Sr21* resistant diploid wheat G3116. Leaves from both genotypes were collected after inoculation with BCCBC and immediately after moving the plants to chambers at 16°C and 24°C (0 h). The statistical analysis for the 0 h control time point is presented in S4A Table, and the combined analysis for one, three and six days after inoculation in S4B Table. Transcript levels were expressed as fold-*ACTIN*. Bars are standard errors of the means. Inoc: inoculated with BCCBC, Mock-Inoc: mock inoculated with water.

We analyzed *CNL1* transcript levels in the four temperature / inoculation combinations described above in resistant accessions G3116 (diploid) and CS*Sr21* (hexaploid). We took samples immediately after moving the plants inoculated with *Pgt* from a greenhouse (~20°C) to growth chambers at 16°C and 24°C (time 0 h, Fig 4). As expected, we found no significant differences in *CNL1* transcript levels between temperatures or inoculations at the control sampling point (S4A Table). The *CNL1* basal transcript levels relative to *ACTIN* were 14.8% higher in CS*Sr21* than in G3116 (*P* = 0.0089, S4A Table).

For the samples collected at 1, 3 and 6 days post inoculation (dpi), we performed a four-way ANOVA for *CNL1* transcript levels including genotype, temperature, inoculation treatment and day after inoculation as factors. We found no significant differences between genotypes (diploid G3116 and hexaploid CS*Sr21*), but detected significantly lower transcript levels in plants grown at 16°C than at 24°C (36.5%, *P <* 0.0001), and in *Pgt* inoculated plants relative to mock-inoculated plants (45.8%, *P <* 0.0001) (Fig 4, S4B Table). A smaller effect was detected among days (*P* = 0.02), but no clear trend was observed.

We used the same RNA samples collected 6 dpi from CS*Sr21* and G3116 described above and primers described in a previous study [16] to quantify the transcript levels of six pathogenesis-related (*PR*) genes (*PR1*, *PR2*, *PR3*, *PR4*, *PR5* and *PR9* = *TaPERO*). For all six *PR* genes, transcript levels were significantly higher (*P* < 0.0001) in *Pgt-*inoculated plants than in mock-inoculated plants (S5 Table and S5 Fig). The overall differences in transcript levels between temperatures were not significant for some *PR* genes (*PR1*, *PR4*, *PR9*) but the interactions between temperature and inoculation were all highly significant in the combined ANOVA (*P* < 0.001, S5 Table). These interactions are clear in S5 Fig, which shows lower *PR* transcript levels at 24°C than at 16°C in the mock-inoculated plants but significantly higher levels in the *Pgt-*inoculated plants at 24°C than at 16°C.

**Fig 5:**
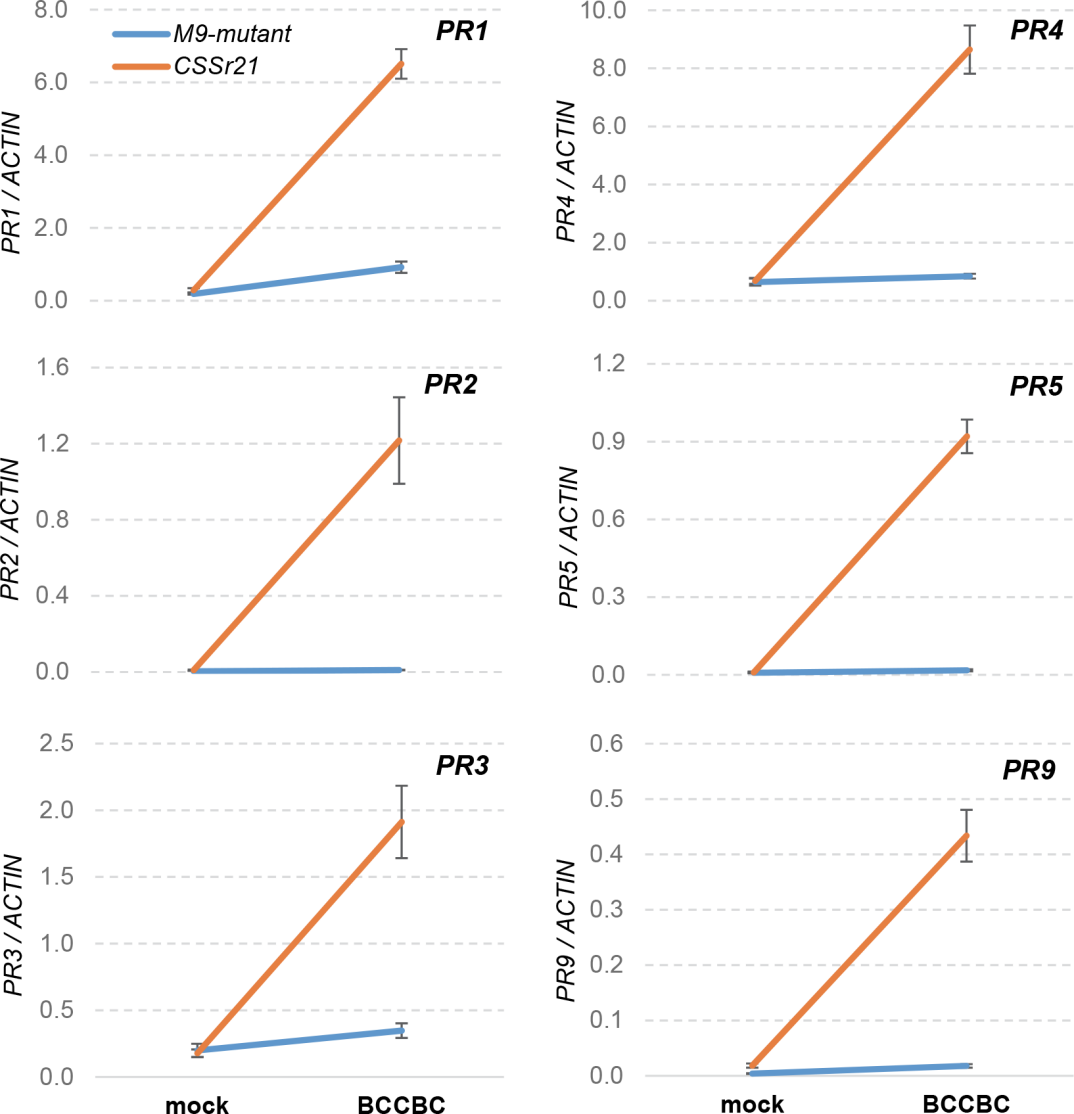
Transcript levels of *PR* genes in the presence and absence of *Sr21*. Transcript levels of *PR* genes (*PR1*, *PR2*, *PR3*, *PR4*, *PR5*, and *PR9*) in inoculated (BCCBC) and mock-inoculated plants with a functional *Sr21* resistance gene (CS*Sr21*) and without it (*sr21*-M9-mutant) grown at 24°C. A strong up-regulation of all *PR* genes was observed only when both *Sr21* and the pathogen were present. Lack of parallelism between lines reflect significant interactions (*P* < 0.001). Values were calculated using the 2^ΔCT^ method relative to *ACTIN* as endogenous control. Error bars indicate standard errors of the means. n = 5. Samples were collected six days post inoculation.

To confirm that *Sr21* was required for the coordinated upregulation of the six *PR* genes at 24°C, we compared the transcript levels of these six genes in the resistant hexaploid line CS*Sr21* and its derived susceptible *sr21*-mutant M9 (Fig 5). Plants from both genotypes were inoculated with race BCCBC or were mock inoculated. For all six *PR* genes, the differences between genotypes, inoculations and the interactions genotype x inoculation were highly significant (*P* < 0.001) in two-way factorial ANOVAs. In all cases, a strong upregulation of all six *PR* genes was observed after inoculation with race BCCBC relative to mock inoculation only in the resistant genotype CS*Sr21* (Fig 5). This result confirmed that the coordinated upregulation of these six *PR* genes was triggered by *Sr21*.

### Effect of temperature and *Sr21* on *Pgt* growth in diploid and hexaploid wheat

**Fig 6:**
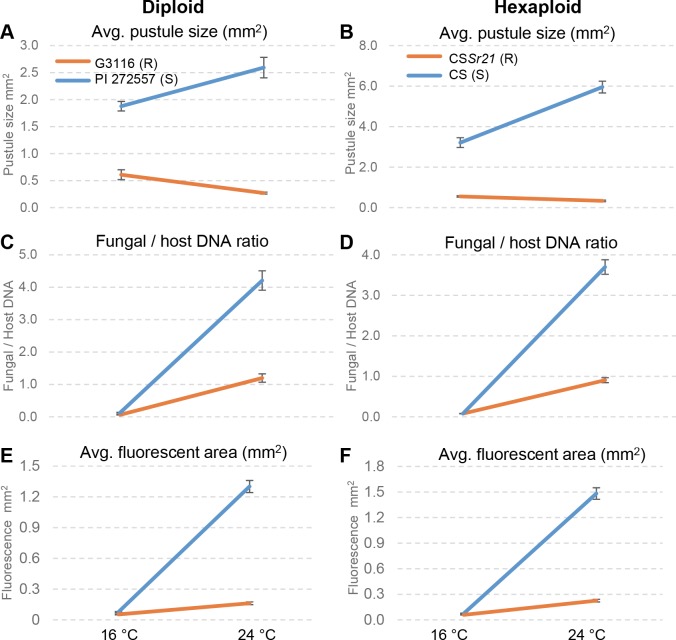
*Pgt* race BCCBC growth at 16°C and 24°C in different genotypes. **(A, C, E)** Diploid wheat. *Sr21* resistant genotype G3116 (orange) and susceptible genotype PI 272557 (blue). **(B, D, E)** Hexaploid wheat. *Sr21* resistant genotype CS*Sr21* (R) (orange) and susceptible genotype CS (S) (blue). **(A, B)** Interaction graphs for average pustule size at 14 dpi n = 6). **(C, D)** Interaction graphs for *Pgt* DNA relative to wheat DNA at 5 dpi (n = 6). **(E, F)** Interaction graphs for average size of individual fungal infection areas estimated by fluorescence microscopy at 5 dpi (n = 6). Lack of parallelism between lines in all graphs reflects significant interactions (*P*<0.0001, S6 Table).

*Sr21* showed higher levels of resistance to BCCBC when plants were grown at 24°C than when grown at 16°C (Figs 6A, 6B and S6). Similar differences were observed before for *Sr21* resistance response to TTKSK at 20°C and 16°C [20]. These observations were confirmed in a three-way ANOVA for average pustule size 14 days post inoculation (dpi) (S6 Table). This analysis showed highly significant (*P* < 0.0001) effects for ploidy level and genotype (presence or absence of *Sr21*), and a very strong interaction between temperature and genotype (*P* < 0.0001). Plants having the *Sr21* resistance gene showed smaller sporulation areas at high temperatures, whereas those without *Sr21* showed significantly larger sporulation areas at high temperatures (Fig 6A and 6B). These opposite effects masked the main effect of temperature for race BCCBC (S6 Table).

We also quantified the differences in BCCBC growth at five dpi by measuring the ratio of *Pgt* DNA relative to wheat DNA (Fig 6C and 6D) and average infection areas using microscopy and a fluorescent dye that stains the pathogen (Figs 6E, 6F and S7). Both methods showed highly significant (*P* < 0.0001) differences between genotypes and between temperatures. As with the sporulation area, we also detected highly significant interactions between genotype and temperature for both methods (*P* < 0.0001, S6 Table), which reflected the larger differences between genotypes at high than at low temperature (Fig 6C–6F). Average areas for sporulation at 14 dpi (TTKSK and BCCBC) and for pathogen growth (BCCBC determined by fluorescence) were larger in the *Sr21-*resistant hexaploid than in the resistant diploid plants, which agrees with a previous report [20].

Fluorescent images of *Pgt* growth in diploid and hexaploid wheat plants lacking *Sr21* at high temperature show a diffuse network of hyphae at the borders of the infected areas, which seems to be expanding without host resistance. By contrast, in the presence of *Sr21*, the infected areas are smaller and their borders are denser (S7 Fig), which suggests that the expansion of the hyphae is facing opposition from the host. At the macroscopic level, a chlorotic halo was observed around the *Pgt* pustules in the *Sr21* resistant reactions, which in some cases resulted in cell death. However, *Pgt* resistance conferred by *Sr21* is mostly associated with a delay in the progression of the disease (partial resistance) rather than with a rapid hypersensitive reaction.

### Haplotype analysis of *CNL1*

We evaluated 114 *T*. *monococcum* and *T*. *urartu* accessions with *Pgt* races MCCFC and BCCBC and confirmed the presence of *Sr21* in 44 of them and its absence in 70. We sequenced the complete *CNL1* gene from these accessions using three pairs of primers (S1 Table and Fig 2A) and identified four susceptible haplotypes (S1 to S4) and five resistant haplotypes (R1 to R5, S8 Table and S8 Fig). The presence of *Sr21* in the resistant diploid wheat accessions is based on inoculation with *Pgt* races TRTTF, TTKSK, TTTTF, QFCSC, and MCCFC (S7 Table).

Of the 44 resistant accessions, 28 were classified as haplotype R1 (MG582649, equal to DV92), six as R2 (MG601519), one as R3 (MG601520), six as R4 (MG601521) and three as R5 (MG601522, S8 Table). The R1 haplotype differed from the other four by one (R2), three (R3), two (R4) and three (R5) amino acid changes, respectively (S8 Fig).

Of the 30 *T*. *monococcum* susceptible accessions, 25 showed identical sequences and were classified as haplotype S1 (MG601523, 90.2% identical at the cDNA level to *CNL1* haplotype R1). S1 is likely a non-functional gene since it carries a frame shift mutation. Surprisingly, polymorphisms between R1 and S1 were concentrated in the CC-NBS domains (87.2% identity) whereas the LRR was more similar to *CNL1* (99.2% identity), which suggests a recombination or conversion event. A phylogenetic analysis including the DNA coding regions of the different NLR genes in the *CNL1* region, and the closest paralogues in *B*. *distachyon* and *H*. *vulgare* showed that the *CNL1* S1 haplotype is more closely related to *cnl7* than to the cluster including the different *CNL1* haplotypes (S9 Fig).

All 40 *T*. *urartu* accessions showed haplotype S2 (MG601524), which was similar to R1 (99.2% identical). This haplotype also includes three accessions classified as *T*. *monococcum* subsp. *aegilopoides* in the NSGC. However, analysis of 12 additional genes showed *T*. *urartu* haplotypes in these three accessions, suggesting that they are misclassified (we counted them as *T*. *monococcum* in the numbers reported above). The S2 haplotype from *T*. *urartu*, and the related haplotypes found in wild tetraploid Zavitan and hexaploid cultivars Chinese Spring and Fielder corresponds to the pseudogene *Cscnl1b* from Fig 1 and shares a 2-bp deletion at positions 3,255 and 3,256 (cDNA coordinates). When this shift mutation is manually corrected, these accessions share 20 amino acid polymorphisms compared to the R1 haplotype (S8 Fig). The cultivated tetraploid Kronos shares 14 of the 20 amino acid polymorphisms found in Chinese Spring, Fielder and Zavitan. Kronos does not have the 2-bp frame shift mutation, but has two different 1-bp deletions at positions 928 (a missing A) and 3,667 (a missing T). The susceptible haplotypes S3 (MG601525) and S4 (MG601526) were very similar to R1 (> 99.9% identical) and included a single accession each (S8 Table). Three accessions from the Balkans, classified as S1 based on their *CNL1* sequence (PI 355538, PI 362610 and PI 377668), showed a resistance reaction when inoculated with race MCCFC but were susceptible to BCCBC. Since *Sr21* is resistant to both BCCBC and MCCFC, this result suggested that these three accessions carry a *Pgt* resistance gene different from *Sr21*.

To develop a diagnostic marker for the *Sr21* resistant haplotypes introgressed into polyploid wheat, we designed primers based on the C1228W diagnostic polymorphism and the T1500R and H1501S polymorphisms that separate *Sr21* resistant and susceptible haplotypes. PCR amplification with primers Sr21TRYF5R5 (S1 Table) at an annealing temperature of 56°C generates a 951-bp fragment when the *T*. *monococcum* haplotypes are present and no amplification in *T*. *urartu*, tetraploid or hexaploid wheat (S10 Fig). Treatment of the amplified PCR products with restriction enzyme *Nsi*I generated two bands of 836 and 115 bp for the *T*. *monococcum* accessions carrying the *Sr21* susceptible haplotypes and a single 951-bp band for the accessions carrying the resistant haplotypes (S10 Fig).

## Discussion

The *T*. *monococcum* genomic region encompassing the *Sr21* resistance gene includes four NLR genes and three pseudogenes (Fig 1). The clustering of NLR genes facilitates the generation of novel variants through recombination and conversion events, increasing genetic variability in resistance genes [27]. Ectopic recombination events can generate deletions and duplications, and these were frequent in the *Sr21* region. We detected several rearrangements between *T*. *monococcum*, hexaploid wheat Chinese Spring and wild tetraploid wheat Zavitan [23] (S1 Fig) that diverged less than one million years ago [28]. A *T*. *monococcum* region of more than 150-kb including *cnl2*, *CNL3*, *cnl4* and *CNL5* was not detected in any of the polyploid species. Similarly, a large region in Chinese Spring between *CsCNL8* and *Cscnl12* was deleted in *T*. *monococcum* and part of it in Zavitan. Finally, a region including *CNL6* and *cnl7* was detected in diploid and tetraploid wheat but was absent in hexaploid wheat. These large differences suggest that this NLR cluster has experienced rapid evolutionary changes. The presence of four related NLR genes and pseudogenes in the colinear region of *Brachypodium distachyon* chromosome 5 (Figs 1 and S9) suggests that this NLR cluster has a long evolutionary history that extends beyond the divergence between the *Triticum* and *Brachypodium* lineages more than 30 million years ago [29].

When we characterized *Sr21* expression, we detected multiple alternative splicing forms of *CNL1*, which differed in the intron structure of the 3’ UTR region (S4 Fig). Alternative splicing forms have been identified in several CC-NBS-LRR genes, including *Pi-ta* in rice [30], *Mla* in barley [31], and *Lr10* [32] in wheat. Complex UTR regions with multiple introns have been also described for other wheat NLR genes involved in resistance to *Pgt* including *Sr35* [12] and *Sr13* [16]. In *Sr21*, we observed differences in the frequencies of the main alternative splicing forms with temperature, but the role of these differences is currently unknown.

Results presented here and in a previous study [20] indicate that *Sr21* is less effective when present in a hexaploid background than in a diploid background. Since the hexaploid *Sr21* gene was introgressed directly from *T*. *monococcum* [21], the encoded proteins are expected to be identical. In addition, we found no significant differences between diploid and hexaploid wheat in *Sr21* transcript levels. Therefore, differences downstream of *Sr21* transcription are likely responsible for the reduced resistance conferred by *Sr21* in hexaploid than in diploid wheat. Reduced stability of the Sr21 protein in hexaploid wheat or reduced compatibility between the *T*. *monococcum* Sr21 protein and some of its downstream *T*. *aestivum* protein interactors are possible explanations. Both hypotheses can explain the stronger upregulation of downstream *PR* genes observed in diploid than in hexaploid *Sr21-*resistant wheat accessions (1.5 fold for *PR2* to 14.6 fold for *PR9*, S5 Table). In both species, the coordinated upregulation of *PR* genes was observed only in the presence of the pathogen and the resistance gene, suggesting that the Sr21 protein needs to be activated through interactions with a *Pgt* effector or a wheat protein modified by *Pgt*.

The stronger resistance response observed at 24°C suggests that temperature modulates some of the involved processes. Elevated growth temperatures have been reported to affect plant resistance to diseases by reducing steady-state levels of resistance protein at high temperatures [33], affecting temperature-sensing NB-LRR proteins [34], or affecting salicylic acid (SA) regulation [35,36]. A similar inhibition of the resistance response by high temperatures has been reported for wheat *Pgt* resistance genes *Sr6*, *Sr10*, *Sr15*, and *Sr17*. By contrast, *Sr13-* [16] and *Sr21-*mediated *Pgt* resistances are more effective at higher temperatures. Both genes also show a coordinated upregulation of the same *PR* genes at high temperatures, which suggests that they may share some common mechanisms.

Although the mechanisms by which *Sr21* and *Sr13* [16] coordinate the upregulation of *PR* genes are currently unknown, information from other species suggests that the NPR1 pathway or WRKY transcription factors may be involved. Previous studies in Arabidopsis have shown that NPR1 interactions with TGA transcription factors play an important role in the regulation of several PR genes [37,38]. This was also observed in barley, in which overexpression of a conserved protein from the stripe rust pathogen that competes with TGA transcription factors for the binding with NPR1, reduced the induction of several *PR* genes in a leaf region adjacent to a bacterial infection [39]. WRKY transcription factors are also interesting candidates because the promoter of several *PR* genes contain W-box elements recognized by these proteins [40,41]. In addition, NLR proteins from barley (MLA) and rice (Pb1) have been shown to interact with WRKY transcription factors to regulate their defense responses [42,43]. It would be interesting to determine if Sr21 or Sr13 can interact with NPR1, WRKY or other transcription factors to coordinate the upregulation of wheat *PR* genes.

The results presented here provide useful information for the utilization of *Sr21* in agriculture. Since *Sr21* is susceptible to some *Pgt* races, it needs to be deployed in combination with other *Pgt* resistance genes or in transgenic cassettes including multiple resistance genes. Given the better Ug99 resistance levels observed in transgenic plants carrying more than one active copy of *Sr21*, it might be valuable to include at least two copies of *Sr21* in the transgenic cassettes. It might be also advisable to avoid combining *Sr21* and *Sr13*, since both genes seem to operate by a similar mechanism involving the activation of multiple *PR* genes at high temperature. Even if *Sr21* and *Sr13* recognize different effectors, the pathogen could bypass both resistance genes simultaneously by attacking a single target if the two genes share a common downstream signaling pathway. Combining genes that operate by different mechanisms may reduce the probability that a single change in the pathogen can defeat multiple pyramided genes [44].

*Sr21* confers only partial resistance to Ug99, but this might be useful in programs that aim to combine multiple partial resistance genes and avoid major all-stage resistance genes, a strategy that has been proposed to increase the durability of wheat resistance to rusts [45]. The deployment of *Sr21* in commercial tetraploid or hexaploid commercial varieties can be accelerated by the diagnostic marker developed in this study (S10 Fig).

## Materials and methods

### Segregating populations and stem rust assays

A total of 7,168 recombinant gametes from two segregating populations were used to construct a high-resolution genetic map of *Sr21*. These populations included 734 F_2_ plants from population PI 272557 × DV92 and 2,850 from population PI 272557 × G3116. Plants with informative recombination events were challenged with races MCCFC (isolate 59KS19) and TTKSK (isolate 04KEN156/04) at the USDA-ARS Cereal Disease Laboratory and with race BCCBC (isolate 09CA115-2) at the University of California, Davis (UCD). Assays of response to race TTKSK were performed at 25°C during the day and 22°C during the night with a 16 h photoperiod (Fig 3) and those for BCCBC at 16°C (low temperature) and 24°C (high temperature). Procedures for inoculation and statistical analyses of infection types were reported previously [20].

### BAC library screening and sequence annotation

A Bacterial Artificial Chromosome (BAC) library from the resistant parent DV92 [22] was used to generate the physical map by chromosome walking. DNAs from the selected BACs were extracted using QIAGEN Large-Construct Kit. BACs were fingerprinted using restriction enzyme *Hind*III, were sequenced using a combination of Illumina Hi Seq2500 at the Beijing Genomic Institute (Sacramento, CA, USA) and WideSeq at Purdue Genomics Core Facility (https://www.purdue.edu/hla/sites/genomics/wideseq-2/), and were assembled using Galaxy [46,47]. We identified and annotated the repetitive elements in the *Sr21* region using the *Triticeae* Repeat Sequence Database (http://wheat.pw.usda.gov/ITMI/Repeats/blastrepeats3.html) and the genes using BLASTN / BLASTX searches in GenBank (http://www.ncbi.nlm.nih.gov/). These three websites were last accessed February 26, 2018.

### EMS mutants screening

Mutant lines were generated by treating 10,000 seeds from the hexaploid wheat line CS*Sr21* (*Sr21* resistance haplotype R1) with 0.8% ethyl methane sulphonate (EMS). Seeds from 1,151 independent M_1_ mutants were harvested, and 25 M_2_ seeds per family were planted and inoculated with *Pgt* race BCCBC (isolate 09CA115-2) at UCD. Twenty-five M_3_ seeds from susceptible M_2_ plants were retested with race MCCFC (isolate 59KS19) at the USDA-ARS Cereal Disease Laboratory.

### 5' and 3' race

Rapid amplification of cDNA ends (RACE) was performed using total RNA extracted from leaves of resistant parent DV92. Both 5' RACE and 3' RACE were performed using the FirstChoice RLM-RACE Kit (Invitrogen) following the manufacturer’s instruction. The PCR products from Nested PCR amplifications were cloned using the TA cloning kit (Invitrogen).

### Wheat transformation and *CNL1* copy number assays

A 10,463-bp genomic DNA fragment including *Sr21* was amplified from DV92 BAC clone 205J7 using Phusion High-Fidelity DNA Polymerase (New England BioLabs Inc.). This fragment, including the complete *Sr21* (5,208 bp) coding region and introns, 2,772 bp upstream from the start codon, and 2,483 bp downstream from the stop codon, was cloned into the binary vector pLC41Hm. The resulting plasmid pLC41HmSr21 was transformed into *Agrobacterium* strain EHA105, and was transformed into the Ug99-susceptible wheat variety Fielder (*CNL1* haplotype S2) at the UC Davis transformation facility (http://ucdptf.ucdavis.edu/). Primers HptmikiF/R developed from the hygromycin resistance gene and primers S21CNL1F5R5 and Sr21TRYF5R5 developed from *CNL1* were used to confirm the presence of transgene (S1 Table). A TaqMan Copy Number Assay was used to estimate the number of copies inserted in every transgenic event as described before [16].

### Alternative splicing

Sequences from the 3' RACE described above and from the transcriptome databases of DV92 and G3116 [24] revealed the presence of several alternative splicing forms at the 3’UTR region of *Sr21*. To determine the number of alternative splicing variants, we first extracted total RNAs from G3116 *T*. *monococcum* plants grown at 16°C and 24°C six days after inoculation. We used these RNAs for 3’RACE reactions and cloned the PCR products using the TA cloning kit (Invitrogen). Eighty colonies from every 3' RACE reaction were PCR-amplified and sequenced using the Sanger method.

### qRT-PCR analysis

Total RNA was extracted from leaves of *Sr21-*resistant diploid *T*. *monococcum* ssp. *aegilopoides* accession G3116 and hexaploid CS*Sr21* using Spectrum Plant Total RNA Kit (Sigma-Aldrich). First strand cDNA was synthesized from 1 µg of total RNA using a High-Capacity cDNA Reverse Transcription Kit (Applied Biosystems). Samples were collected from plants grown under the four treatments resulting from the combination of two temperatures (16°C or 24°C) and two inoculation treatments (*Pgt* race BCCBC or mock inoculation). Samples were collected immediately after inoculation (0 h) and 1, 3 and 6 days post inoculation (dpi). We quantified *Sr21* expression using primers Sr21qRTF1R1 (S1 Table). The same cDNA samples were used to quantify the transcript levels of six pathogenesis-related (*PR*) genes (*PR1*, *PR2*, *PR3*, *PR4*, *PR5* and *PR9* = *TaPERO*) using primers described before [16].

We performed the qRT-PCR reactions on an ABI 7500 Fast Real-Time PCR System (Applied Biosystems) using Fast SYBR GREEN Master Mix. Transcript levels were expressed as fold-*ACTIN* levels (the number of molecules in the target / the number of *ACTIN* molecules) using the 2^ΔCT^ method as described before [48]. We calculated the significance of the differences in expression levels using factorial ANOVAs and the SAS program version 9.4.

### Haplotype analysis of *CNL1*

From a previous study [49], we selected 44 *T*. *monococcum* accessions (28 cultivated and 16 wild type) resistant to races MCCFC and TTKSK and susceptible to races TTTTF, TRTTF and QFCSC, which were previously postulated to carry only *Sr21* (S7 Table). We also selected 30 accessions of *T*. *monococcum* and 40 from *T*. *urartu* that were susceptible to all five races (S7 Table). Seeds were obtained from the U.S. Department of Agriculture National Small Grains Collection (NSGC).

All *T*. *monococcum* and *T*. *urartu* accessions were re-evaluated with races MCCFC (isolate 59KS19) and BCCBC (isolate 09CA115-2) (at 24°C) for this study. We also confirmed the absence of *Sr35* with the diagnostic marker developed from the cloned *Sr35* gene [12]. Seeds from three *T*. *monococcum* accessions showed heterogeneous resistance reactions to MCCFC (PI 427971, PI 119422 and PI 225164) and were listed twice in S8 Table (as–S and–R) resulting in a total of 74 *T*. *monococcum* accessions. The sequenced genomes of diploid wheat *T*. *urartu* (http://plants.ensembl.org/Triticum_urartu/Info/Index), tetraploid wheat Zavitan (https://wheat.pw.usda.gov/GG3/wildemmer), and hexaploid wheat Chinese Spring RefSeq v1 (https://urgi.versailles.inra.fr/blast_iwgsc/blast.php) were used to detect the corresponding *CNL1* alleles.

## Supporting information

S1 Fig BACs in *Sr21* region. **(A)** Proximal chromosome walk group: 22 BACs from the region proximal to *Sr21*. All BACs were selected from the DV92 BAC library and fingerprinted with restriction enzyme *Hind*III [22]. Markers *CJ961291* and *179C18F7R7* delimit a 405-kb candidate region for *Sr21* (blue shaded square). **(B)** Distal chromosome walk group: 17 *T*. *monococcum* BACs from the region distal to *Sr21*. In both panels, BACs in the minimum tilling path that were sequenced are indicated in red. Colored ovals represent markers used for chromosome walking, whereas colored arrows represent genes identified in the BAC sequences.
(PDF)

S2 Fig Transcript levels and copy number of *CNL1* in transgenic T_0_ plants. Transcript levels of *CNL1* in 13 transgenic T_0_ plants based on three technical replicates from a single plant. Numbers below rectangles indicate the copy number of transgenes based on the TaqMan copy number assay. Transcript levels are expressed as fold-*ACTIN* using the 2^ΔCT^ method. Fielder has a non-functional copy of *CNL1*.
(PDF)

S3 Fig Correlation between resistance to Ug99 and number of *CNL1* copies in transgenic wheat plants. **(A)** Six T_1_ plants from family T_1_Sr21-03 inoculated with *Pgt* race TTKSK (Ug99). **(B)** Six T_1_ plants from family T_1_Sr21-04 inoculated with TTKSK. The first row of numbers below the figure indicates the average pustule size estimated using the image analysis software ASSESS v.2.0 (n = 3). The second row of numbers is the estimated number of *CNL1* copies in the transgenic plants based on a TaqMan copy number assay relative to CS*Sr21*. Pustule size was inversely correlated with the estimated number of *CNL1* copies (*R* = -0.92, *P* < 0.0001).
(PDF)

S4 Fig *CNL1* structure and alternative splice sites in 3’ UTR region. Yellow rectangles indicate coding regions and dotted blue lines introns. Gray rectangles represent 3’ and 5’ untranslated regions (UTR). The transcriptome databases of DV92 and G3116 and sequencing of multiple clones from 3' RACE reactions revealed ten alternative splice variants of *CNL1* 3’UTR (*CNL1*-1 to *CNL1*-10).
(PDF)

S5 Fig Interaction between temperature and inoculation with race BCCBC. Transcript levels of *Pathogenesis Related* genes *PR1*, *PR2*, *PR3*, *PR4*, *PR5*, and *PR9* (*TaPERO*). Diploid *T*. *monococcum* (G3116) n = 4 and hexaploid *T*. *aestivum* (CS*Sr21*) n = 3. Values were calculated using the 2^ΔCT^ method relative to *ACTIN* endogenous control (scales are comparable across genotypes). Error bars indicate standard errors of the means. Lack of parallelism between lines in all graphs indicate significant interactions (*P* < 0.0001, S5 Table).
(PDF)

S6 Fig Interaction between temperature and sporulation area. **(A-B)** Inoculation with *Pgt* race BCCBC at 16°C **(A)** and 24°C **(B)**. The hexaploid susceptible line is Chinese Spring (CS, “-”) and the resistant line is CS*Sr21* (“*Sr21*”). The diploid susceptible line is *T*. *monococcum* PI 272557 (“-”) and the resistant line is G3116 (“*Sr21*”). Similar differences were observed before for *Sr21* resistance to TTKSK at 16°C and 20°C [20].
(PDF)

S7 Fig *Pgt* infection areas visualized by fluorescent staining. **(A-D)** Race BCCBC growth at 16°C. **(A-B)** Hexaploid wheat, **(C-D)** diploid wheat. **(E-H)** Race BCCBC growth at 24°C. **(E-F)** Hexaploid wheat, **(G-H)** diploid wheat. CS*Sr21* and G3116 are the hexaploid and diploid resistant lines carrying *Sr21*, respectively. CS and PI 272557 are the hexaploid and diploid susceptible lines, respectively. Pictures were taken five days post inoculation (dpi). Infected leaves were cleared with KOH and stained with WGA-FITC. Interaction graphs are presented in Fig 6E and 6F, and statistical analyses in S6 Table. Scale bars represent 500 μm.
(PDF)

S8 Fig *CNL1* haplotypes. ***T*. *monococcum* Ug99 resistant (R) and susceptible (S) accessions**. Four bottom lines are *CNL1* closest homologs from *T*. *urartu* and A-genome of polyploid wheat. Red highlight indicates alleles present in a single haplotype. The first part of haplotype S1 is too divergent and is not presented here.
(PDF)

S9 Fig Phylogenetic tree of NLR genes related to *CNL1*. Neighbor-Joining tree analysis including *CNL1* haplotypes, linked *CNL3*, *CNL5* and *cnl7* genes (blue squares), and the closest predicted genes from *T*. *urartu* (T.u.), *T*. *dicoccoides* (Tdic), *Triticum aestivum* (Traes), *Hordeum vulgare* (HORVU), and *Brachypodium distachyon* (Bradi). Coding DNA sequences were aligned with muscle as implemented in Mega 7, and phylogenetic trees were then generated using the pair-wise deletion method (bootstrap confidence values based on 1000 iterations).
(PDF)

S10 Fig *Sr21* diagnostic PCR marker Sr21TRYF5R5 digested with *Nsi*I. The undigested 951-bp PCR products (black arrow) are present in genotypes DV92 (R1), G3116 (R3), PI 306540 (R2), CS*Sr21* (R1), PI 427971-R (R4) and PI 427796 (R5) carrying the different *Sr21* resistant haplotypes. A digested band of 836-bp (yellow arrowhead, 115 bp band out of the gel) was detected in *T*. *monococcum* genotypes PI 427971-S and PI 538540 that carry the *Sr21* susceptible haplotypes S3 or S4. No amplification product was detected with these primers for susceptible genotypes of *T*. *monococcum* PI 272557 (S1), *T*. *urartu* PI 428227 (S2), PI 428183 (S2), and the related susceptible haplotypes present in Kronos, Fielder and Chinese Spring (CS) (S8 Fig and S8 Table).
(PDF)

S1 Table Primers used in this study. Primers for high density genetic map, screening of the *T*. *monococcum* BAC library, haplotype analysis, mutant screening, marker-assisted selection (MAS), expression analysis, 3’ and 5’ RACE, cloning and screening for transgenic studies, and copy number assays.
(PDF)

S2 Table Estimated copy number of transgenic insertions. Number of insertions in each transgenic event based on T_1_ plants and a TaqMan copy number assay of the transgenic plants relative to CS*Sr21* (1 copy).
(PDF)

S3 Table Alternative splicing forms. Alternative splicing forms were identified from multiple 3' RACE reactions (total RNAs of G3116 from 24°C / Inoculated / 6 d, 24°C / Mock-inoculated / 6 d, 16°C / Inoculated / 6 d and 16°C / Mock-inoculated / 6 d). Eighty colonies (using TA-cloning) from every 3' RACE reaction were PCR amplified and sequenced using the Sanger method. Eighty clones were analyzed for each of the four conditions. For the χ^2^ analysis, the five less frequent alternative splice forms were merged (*CNL1-*3, *CNL1-*4, *CNL1-*7, *CNL1-*8, *CNL1-*9, and *CNL1-*10).
(PDF)

S4 Table *CNL1* expression levels. Transcript levels at two temperatures (16°C and 24°C), two *Pgt* treatments (inoculated and mock-inoculated), and four collection time points after inoculation (0 hour, 1 dpi, 3 dpi and 6 dpi). **(A)** Three-way ANOVA at time 0 immediately after inoculation and transfer from greenhouse at 20°C to the chambers at 16°C and 24°C. **(B)** Four-way ANOVA at 1, 3 and 6 dpi.
(PDF)

S5 Table Three-way ANOVA. *P* values of transcript levels of *PR* genes in CS*Sr21* and G3116 plants 6 dpi with *Pgt* race BCCBC or mock-inoculated at high (24°C) and low (16°C) temperatures. CS*Sr21* and G3116 indicate *P* values for the individual two-way ANOVAS. The last row indicates the ratio between the diploid and hexaploid average expression of the *PR* gene in the *Pgt* inoculated plants at 24°C.
(PDF)

S6 Table Pathogen growth at different temperatures in susceptible and *Sr21-*resistant diploid and hexaploid wheat. Sporulation size was determined at 14 dpi, whereas *Pgt*/host DNA ratio and area of fungal growth detected by fluorescence were determined at 5 dpi. Experiments with BCCBC were performed at 16°C and 24°C and those for TTKSK were performed at 16°C and 20°C and were reported before [20]. Six replications were used per treatment combination, with the exception of sporulation size for race TTKSK where only three replications were used. Normality of residuals of the transformed data was confirmed using Shapiro-Wilk test.
(PDF)

S7 Table Infection types. Infection types in diploid accessions (*Triticum monococcum* subsp. *aegilopoides*, *Triticum monococcum* subsp. *monococcum* and *Triticum urartu*) to five *Pgt* races TRTTF, TTKSK, TTTTF, QFCSC, and MCCFC. Infection types shown here were based on the previous study [49].
(PDF)

S8 Table Haplotypes of *CNL1*. *CNL1* haplotypes among a collection of 74 *T*. *monococcum* and 40 *T*. *urartu* accessions.
(PDF)
