# Supplementary material for: Identification and characterization of wheat stem rust resistance gene Sr21 effective against the Ug99 race group at high temperature
Source: PLoS Genet. 2018 Apr 3;14(4):e1007287. doi: 10.1371/journal.pgen.1007287 (PMC5882135; doi:10.1371/journal.pgen.1007287)
Supplement: S1 Fig — (A) Proximal chromosome walk group: 22 BACs from the region proximal to Sr21. All BACs were selected from the DV92 BAC library and fingerprinted with restriction enzyme HindIII [22]. Markers CJ961291 and 179C18F7R7 delimit a 405-kb candidate region for Sr21 (blue shaded square). (B) Distal chromosome walk group: 17 T. monococcum BACs from the region distal to Sr21. In both panels, BACs in the minimum tilling path that were sequenced are indicated in red. Colored ovals represent markers used for chromosome walking, whereas colored arrows represent genes identified in the BAC sequences. (PDF) [file pgen.1007287.s001.pdf]

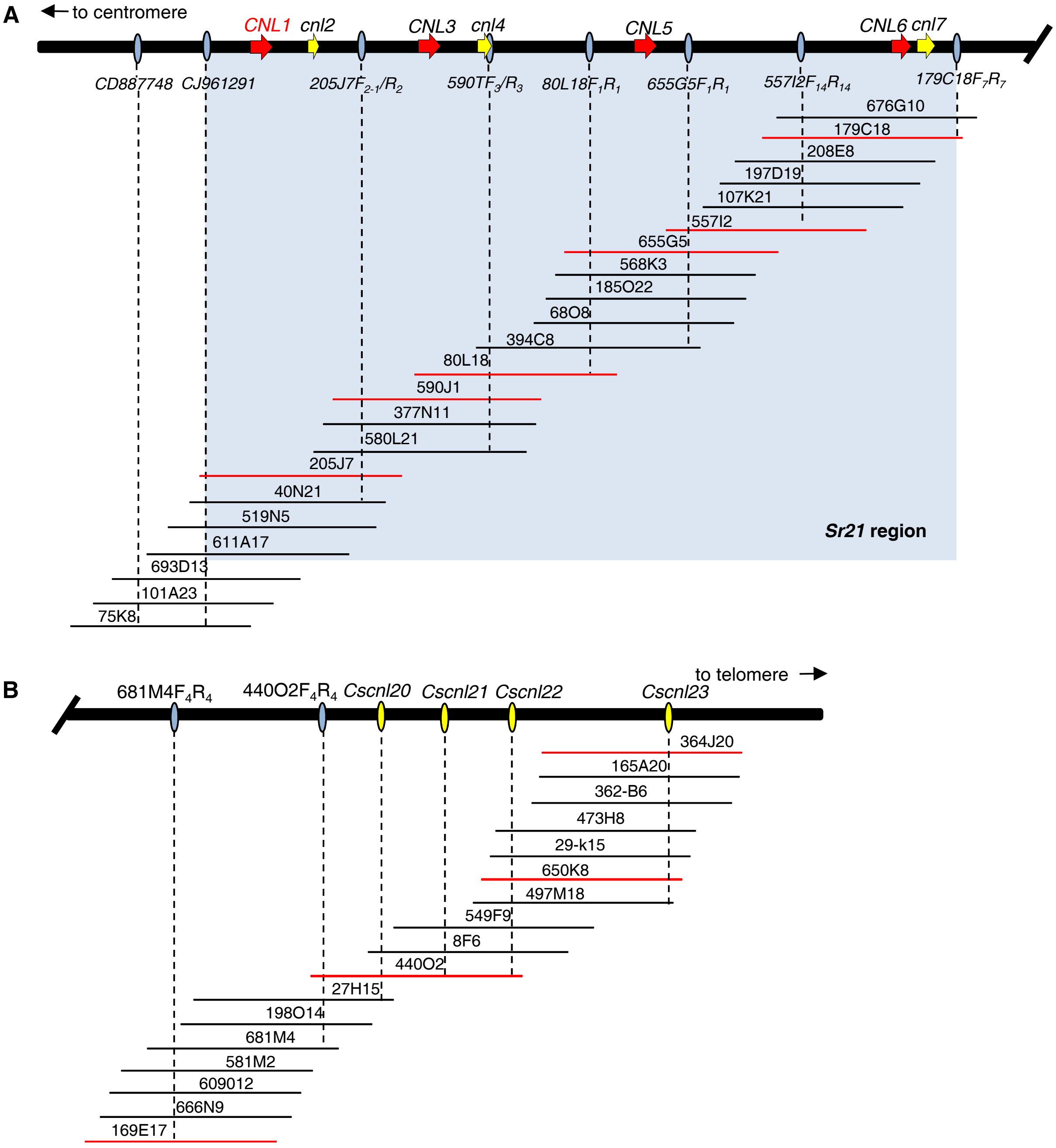

S1 Fig. BACs in *Sr21* region. **(A)** Proximal chromosome walk group: 22 BACs from the region proximal to *Sr21*. All BACs were selected from the DV92 BAC library and fingerprinted with restriction enzyme *Hind*III [22]. Markers *CJ961291* and *179C18F7R7* delimit a 405-kb candidate region for *Sr21* (blue shaded square). **(B)** Distal chromosome walk group: 17 *T. monococcum* BACs from the region distal to *Sr21*. In both panels, BACs in the minimum tilling path that were sequenced are indicated in red. Colored ovals represent markers used for chromosome walking, whereas colored arrows represent genes identified in the BAC sequences.
