# Supplementary material for: Identification and characterization of wheat stem rust resistance gene Sr21 effective against the Ug99 race group at high temperature
Source: PLoS Genet. 2018 Apr 3;14(4):e1007287. doi: 10.1371/journal.pgen.1007287 (PMC5882135; doi:10.1371/journal.pgen.1007287)
Supplement: S2 Fig — Transcript levels of CNL1 in 13 transgenic T0 plants based on three technical replicates from a single plant. Numbers below rectangles indicate the copy number of transgenes based on the TaqMan copy number assay. Transcript levels are expressed as fold-ACTIN using the 2ΔCT method. Fielder has a non-functional copy of CNL1. (PDF) [file pgen.1007287.s002.pdf]

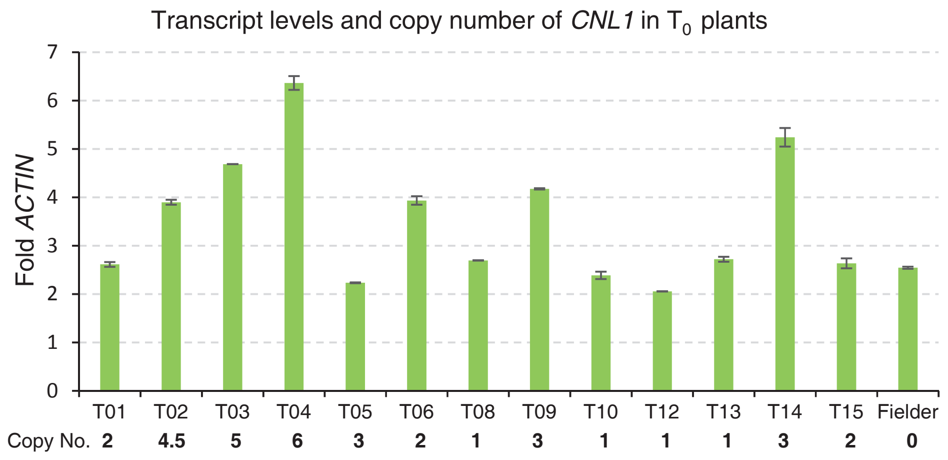

S2 Fig. Transcript levels and copy number of *CNL1* in transgenic T_0 plants. Transcript levels of *CNL1* in 13 transgenic T_0 plants based on three technical replicates from a single plant. Numbers below rectangles indicate the copy number of transgenes based on the TaqMan copy number assay. Transcript levels are expressed as fold-*ACTIN* using the $2^{\Delta CT}$ method. Fielder has a non-functional copy of *CNL1*.
