# Supplementary material for: Identification and characterization of wheat stem rust resistance gene Sr21 effective against the Ug99 race group at high temperature
Source: PLoS Genet. 2018 Apr 3;14(4):e1007287. doi: 10.1371/journal.pgen.1007287 (PMC5882135; doi:10.1371/journal.pgen.1007287)
Supplement: S3 Fig — (A) Six T1 plants from family T1Sr21-03 inoculated with Pgt race TTKSK (Ug99). (B) Six T1 plants from family T1Sr21-04 inoculated with TTKSK. The first row of numbers below the figure indicates the average pustule size estimated using the image analysis software ASSESS v.2.0 (n = 3). The second row of numbers is the estimated number of CNL1 copies in the transgenic plants based on a TaqMan copy number assay relative to CSSr21. Pustule size was inversely correlated with the estimated number of CNL1 copies (R = -0.92, P < 0.0001). (PDF) [file pgen.1007287.s003.pdf]

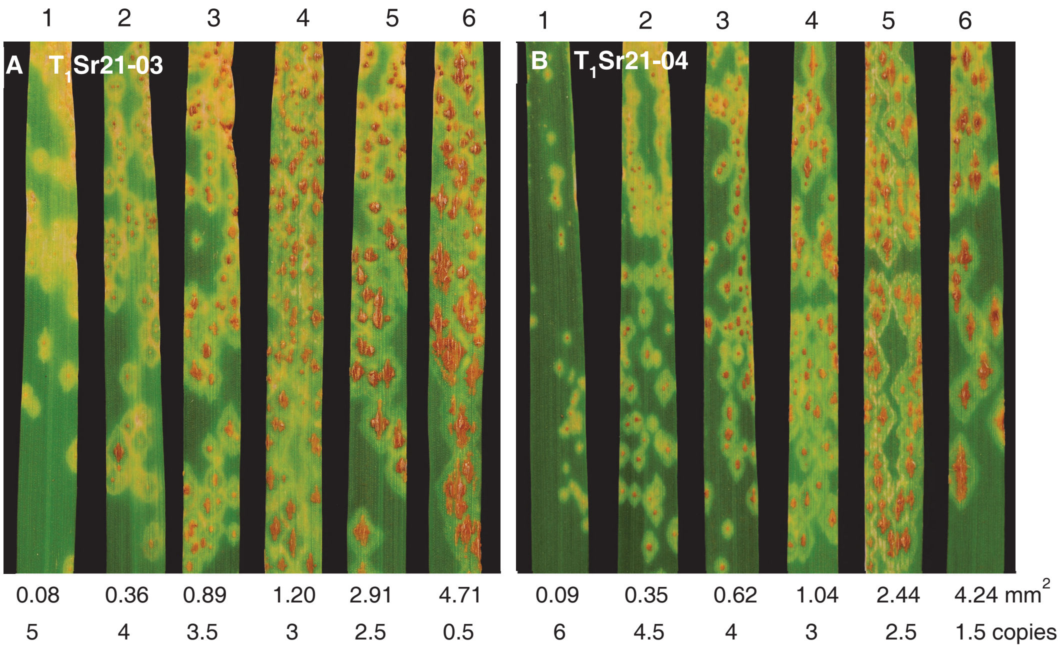

S3 Fig. Correlation between resistance to Ug99 and number of *CNL1* copies in transgenic wheat plants. (A) Six T_1 plants from family T_1Sr21-03 inoculated with *Pgt* race TTKSK (Ug99). **(B)** Six T_1 plants from family T_1Sr21-04 inoculated with TTKSK. The first row of numbers below the figure indicates the average pustule size estimated using the image analysis software ASSESS v.2.0 (n=3). The second row of numbers is the estimated number of *CNL1* copies in the transgenic plants based on a TaqMan copy number assay relative to CS*Sr21*. Pustule size was inversely correlated with the estimated number of *CNL1* copies (R= -0.92, P < 0.0001).
