# Supplementary material for: Identification and characterization of wheat stem rust resistance gene Sr21 effective against the Ug99 race group at high temperature
Source: PLoS Genet. 2018 Apr 3;14(4):e1007287. doi: 10.1371/journal.pgen.1007287 (PMC5882135; doi:10.1371/journal.pgen.1007287)
Supplement: S4 Fig — Yellow rectangles indicate coding regions and dotted blue lines introns. Gray rectangles represent 3’ and 5’ untranslated regions (UTR). The transcriptome databases of DV92 and G3116 and sequencing of multiple clones from 3' RACE reactions revealed ten alternative splice variants of CNL1 3’UTR (CNL1-1 to CNL1-10). (PDF) [file pgen.1007287.s004.pdf]

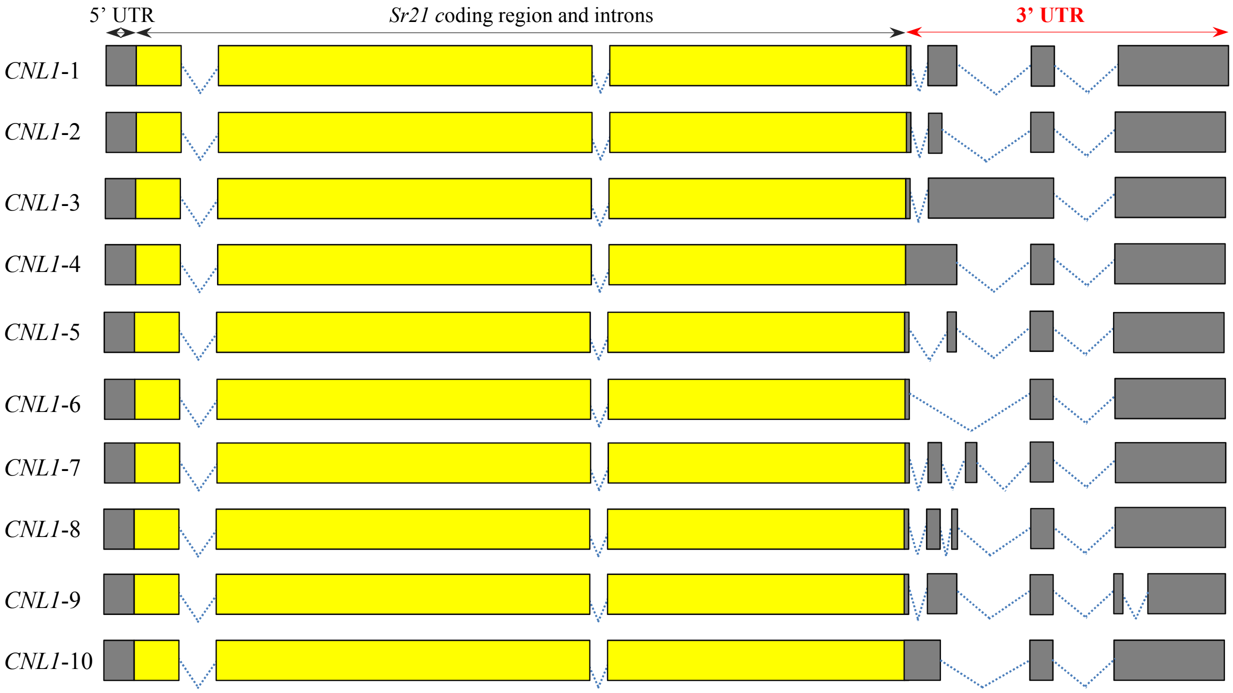

S4 Fig. *CNL1* structure and alternative splice sites in 3' UTR region. Yellow rectangles indicate coding regions and dotted blue lines introns. Gray rectangles represent 3' and 5' untranslated regions (UTR). The transcriptome databases of DV92 and G3116 and sequencing of multiple clones from 3' RACE reactions revealed ten alternative splice variants of *CNL1* 3'UTR (*CNL1*-1 to *CNL1*-10).
