# Supplementary material for: Identification and characterization of wheat stem rust resistance gene Sr21 effective against the Ug99 race group at high temperature
Source: PLoS Genet. 2018 Apr 3;14(4):e1007287. doi: 10.1371/journal.pgen.1007287 (PMC5882135; doi:10.1371/journal.pgen.1007287)
Supplement: S5 Fig — Transcript levels of Pathogenesis Related genes PR1, PR2, PR3, PR4, PR5, and PR9 (TaPERO). Diploid T. monococcum (G3116) n = 4 and hexaploid T. aestivum (CSSr21) n = 3. Values were calculated using the 2ΔCT method relative to ACTIN endogenous control (scales are comparable across genotypes). Error bars indicate standard errors of the means. Lack of parallelism between lines in all graphs indicate significant interactions (P < 0.0001, S5 Table). (PDF) [file pgen.1007287.s005.pdf]

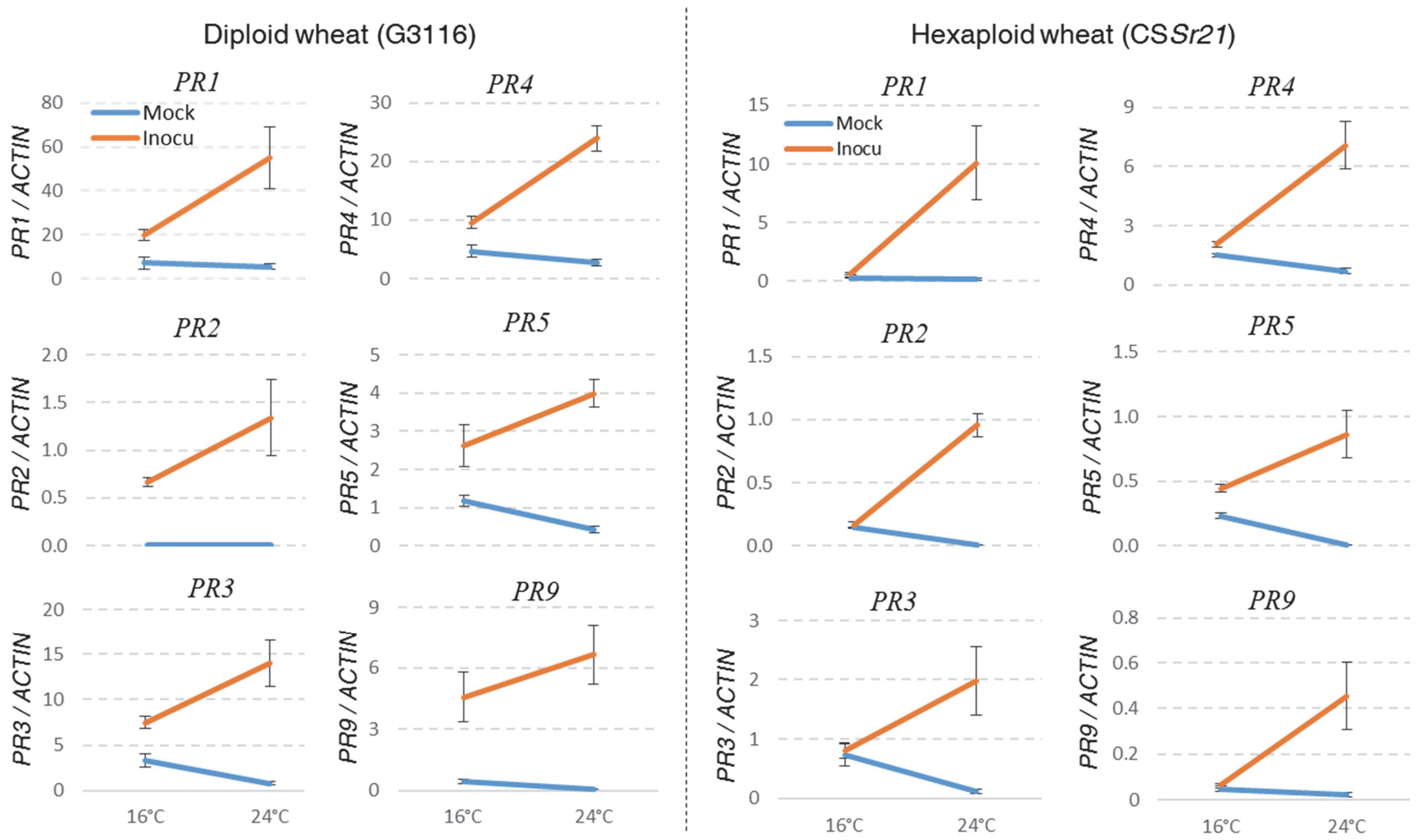

S5 Fig. **Interaction between temperature and inoculation with race BCCBC**. Transcript levels of *Pathogenesis Related* genes *PR1*, *PR2*, *PR3*, *PR4*, *PR5*, and *PR9* (*TaPERO*). Diploid *T. monococcum* (G3116) n=4 and hexaploid *T. aestivum* (CS*Sr21*) n=3. Values were calculated using the $2^{\Delta C_T}$ method relative to *ACTIN* endogenous control (scales are comparable across genotypes). Error bars indicate standard errors of the means. Lack of parallelism between lines in all graphs indicate significant interactions ($P < 0.0001$, S5 Table).
