# Supplementary material for: Identification and characterization of wheat stem rust resistance gene Sr21 effective against the Ug99 race group at high temperature
Source: PLoS Genet. 2018 Apr 3;14(4):e1007287. doi: 10.1371/journal.pgen.1007287 (PMC5882135; doi:10.1371/journal.pgen.1007287)
Supplement: S6 Fig — (A-B) Inoculation with Pgt race BCCBC at 16°C (A) and 24°C (B). The hexaploid susceptible line is Chinese Spring (CS, “-”) and the resistant line is CSSr21 (“Sr21”). The diploid susceptible line is T. monococcum PI 272557 (“-”) and the resistant line is G3116 (“Sr21”). Similar differences were observed before for Sr21 resistance to TTKSK at 16°C and 20°C [20]. (PDF) [file pgen.1007287.s006.pdf]

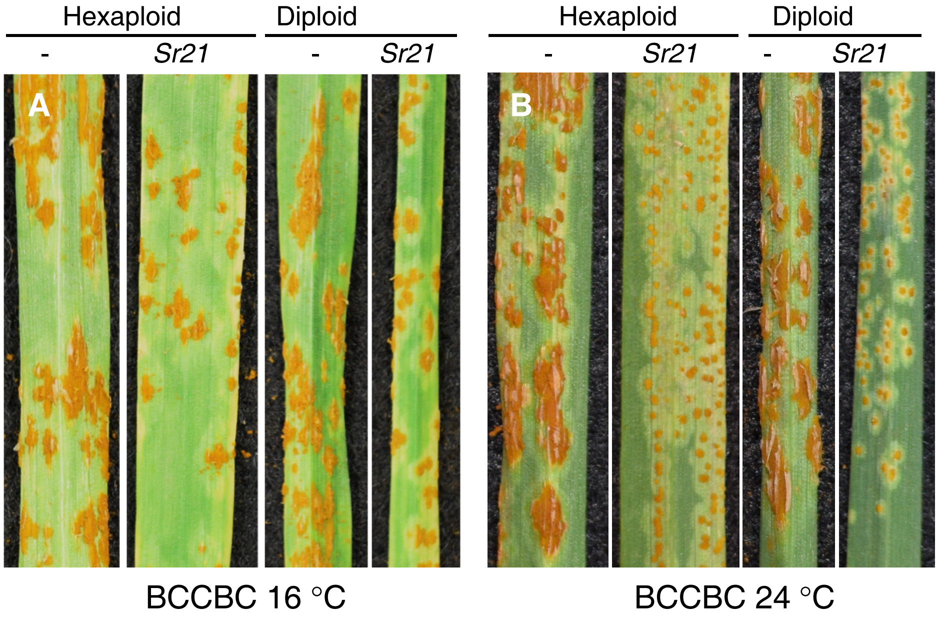

S6 Fig. Interaction between temperature and sporulation area. (A-B) Inoculation with *Pgt* race BCCBC at 16 °C **(A)** and 24 °C **(B)**. The hexaploid susceptible line is Chinese Spring (CS, "-") and the resistant line is CS*Sr21* ("*Sr21*"). The diploid susceptible line is *T. monococcum* PI 272557 ("-") and the resistant line is G3116 ("*Sr21*"). Similar differences were observed before for *Sr21* resistance to TTKSK at 16 ° C and 20 °C [20].
