# Supplementary material for: Identification and characterization of wheat stem rust resistance gene Sr21 effective against the Ug99 race group at high temperature
Source: PLoS Genet. 2018 Apr 3;14(4):e1007287. doi: 10.1371/journal.pgen.1007287 (PMC5882135; doi:10.1371/journal.pgen.1007287)
Supplement: S7 Fig — (A-D) Race BCCBC growth at 16°C. (A-B) Hexaploid wheat, (C-D) diploid wheat. (E-H) Race BCCBC growth at 24°C. (E-F) Hexaploid wheat, (G-H) diploid wheat. CSSr21 and G3116 are the hexaploid and diploid resistant lines carrying Sr21, respectively. CS and PI 272557 are the hexaploid and diploid susceptible lines, respectively. Pictures were taken five days post inoculation (dpi). Infected leaves were cleared with KOH and stained with WGA-FITC. Interaction graphs are presented in Fig 6E and 6F, and statistical analyses in S6 Table. Scale bars represent 500 μm. (PDF) [file pgen.1007287.s007.pdf]

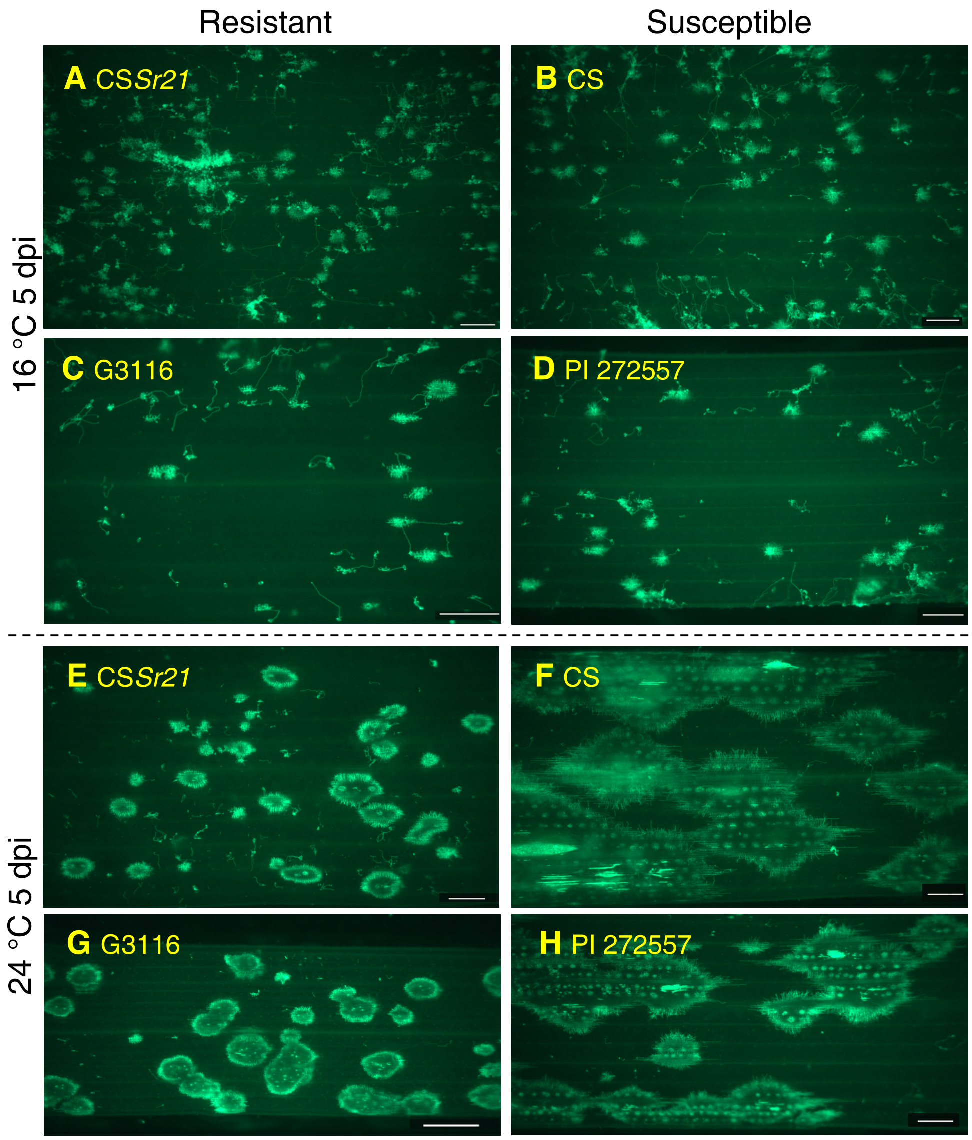

S7 Fig. *Pgt* infection areas visualized by fluorescent staining. (A-D) Race BCCBC growth at 16 °C. **(A-B)** Hexaploid wheat, **(C-D)** diploid wheat. **(E-H)** Race BCCBC growth at 24 °C. **(E-F)** Hexaploid wheat, **(G-H)** diploid wheat. CS*Sr21* and G3116 are the hexaploid and diploid resistant lines carrying *Sr21*, respectively. CS and PI 272557 are the hexaploid and diploid susceptible lines, respectively. Pictures were taken five days post inoculation (dpi). Infected leaves were cleared with KOH and stained with WGA-FITC. Interaction graphs are presented in Fig. 6e-f, and statistical analyses in S6 Table. Scale bars represent 500 µm.
