# Supplementary material for: Identification and characterization of wheat stem rust resistance gene Sr21 effective against the Ug99 race group at high temperature
Source: PLoS Genet. 2018 Apr 3;14(4):e1007287. doi: 10.1371/journal.pgen.1007287 (PMC5882135; doi:10.1371/journal.pgen.1007287)
Supplement: S8 Fig — T. monococcum Ug99 resistant (R) and susceptible (S) accessions. Four bottom lines are CNL1 closest homologs from T. urartu and A-genome of polyploid wheat. Red highlight indicates alleles present in a single haplotype. The first part of haplotype S1 is too divergent and is not presented here. (PDF) [file pgen.1007287.s008.pdf]

Haplotype	CC domain			NBS domain																						
	7	23	75	143	182	221	242	287	310	344	383	431	436	451	548	553	581	631	646	669	750	780	867	880	894	977
**R1**	T	S	G	R	S	Y	S	F	S	W	T	V	S	W	A	V	T	V	S	A	V	L	N	I	Y	V
**R2**	T	S	G	R	S	Y	S	F	S	W	T	V	S	W	A	V	T	V	S	A	V	L	N	I	Y	V
**R3**	T	S	G	R	S	Y	S	F	S	W	T	V	S	W	V	V	T	V	S	A	V	L	N	I	Y	V
**R4**	T	S	G	R	S	Y	S	F	S	W	T	V	S	W	V	V	T	V	S	A	V	L	N	I	Y	V
**R5**	T	S	G	R	S	Y	S	F	S	W	T	V	S	W	V	V	T	V	S	A	V	L	N	I	Y	V
**S1**	-	-	-	-	-	-	-	-	-	-	-	-	-	-	-	-	-	-	-	-	-	-	-	-	-	-
**S2**	T	S	D	H	N	S	S	F	S	W	T	A	S	R	V	V	A	E	S	V	V	L	N	I	C	V
**S3**	T	S	G	R	S	Y	S	F	S	S	T	V	S	W	A	I	T	V	I	A	V	L	N	I	Y	V
**S4**	T	S	G	R	S	Y	S	F	S	W	T	V	S	W	A	V	T	V	S	A	V	L	N	I	Y	V
**Zacnl1b**	M	S	D	H	N	S	S	F	S	W	T	V	S	R	V	V	A	E	S	A	V	L	N	I	Y	V
**Cscnl1b**	T	S	D	H	N	S	F	L	S	W	T	V	S	R	V	V	A	E	S	A	V	M	D	I	Y	I
**Kronos**	T	N	G	R	N	Y	S	F	-1bp	W	S	V	N	W	V	V	T	E	S	A	A	L	N	T	Y	V
***T.u.* G1812**	T	S	D	H	N	S	S	F	S	W	T	A	S	R	V	V	A	E	S	A	V	L	N	I	C	V

Haplotype	LRR domain																											Phe.
	984	998	1020	1062	1086	1182	1194	1206	1228	1223	1251	1293	1384	1445	1446	1458	1482	1488	1500	1501	1510	1515	1518	1521	1586	1592	1620	
**R1**	V	T	R	H	I	Q	T	T	W	S	M	P	L	E	S	S	V	Q	T	H	R	G	A	G	L	Y	R	Re
**R2**	V	T	R	H	I	Q	T	T	W	S	M	P	L	E	S	S	V	Q	T	H	R	G	A	G	L	Y	S	Re
**R3**	V	T	R	H	I	Q	T	T	W	S	T	P	L	E	S	S	V	Q	T	H	R	G	A	G	L	Y	L	Re
**R4**	V	T	R	H	I	Q	T	T	W	S	T	P	L	E	S	S	V	Q	T	H	R	G	A	G	L	Y	R	Re
**R5**	V	T	R	H	I	Q	T	I	W	S	T	P	L	E	S	S	V	Q	T	H	R	G	A	G	L	Y	R	Re
**S1**	-	-	-	-	-	-	-	-	C	S	T	P	L	-5	bp	S	V	Q	T	H	R	V	A	G	L	Y	R	Su
**S2**	V	M	R	R	-2bp	Q	M	T	C	S	T	Q	L	E	S	Y	A	E	R	S	G	G	A	W	L	S	R	Su
**S3**	V	T	R	H	I	Q	T	T	C	S	M	P	L	E	S	S	V	Q	T	H	R	G	A	G	L	Y	R	Su
**S4**	V	T	R	H	I	Q	T	T	C	S	M	P	L	E	S	S	V	Q	T	H	R	G	A	G	P	Y	R	Su
**Zacnl1b**	A	T	C	R	-2bp	Q	M	T	C	S	T	Q	F	E	S	Y	A	E	R	S	G	G	A	W	L	S	R	Su
**Cscnl1b**	A	T	R	R	-2bp	Q	M	T	C	S	T	Q	L	E	S	Y	A	E	R	S	G	G	T	W	L	S	R	Su
**Kronos**	V	T	R	H	I	P	M	T	C	-1bp	T	Q	L	E	S	Y	A	E	R	S	G	G	A	W	L	S	R	Su
***T.u.* G1812**	V	M	R	H	-2bp	Q	M	T	C	S	T	Q	L	E	S	Y	A	E	R	S	G	G	A	W	L	S	R	Su

S8 Fig. *CNL1* haplotypes. *T. monococcum* Ug99 resistant (R) and susceptible (S) accessions. Four bottom lines are *CNL1* closest homologs from *T. urartu* and A-genome of polyploid wheat. Red highlight indicates alleles present in a single haplotype. The first part of haplotype S1 is too divergent and is not presented here.
