# Supplementary material for: Identification and characterization of wheat stem rust resistance gene Sr21 effective against the Ug99 race group at high temperature
Source: PLoS Genet. 2018 Apr 3;14(4):e1007287. doi: 10.1371/journal.pgen.1007287 (PMC5882135; doi:10.1371/journal.pgen.1007287)
Supplement: S9 Fig — Neighbor-Joining tree analysis including CNL1 haplotypes, linked CNL3, CNL5 and cnl7 genes (blue squares), and the closest predicted genes from T. urartu (T.u.), T. dicoccoides (Tdic), Triticum aestivum (Traes), Hordeum vulgare (HORVU), and Brachypodium distachyon (Bradi). Coding DNA sequences were aligned with muscle as implemented in Mega 7, and phylogenetic trees were then generated using the pair-wise deletion method (bootstrap confidence values based on 1000 iterations). (PDF) [file pgen.1007287.s009.pdf]

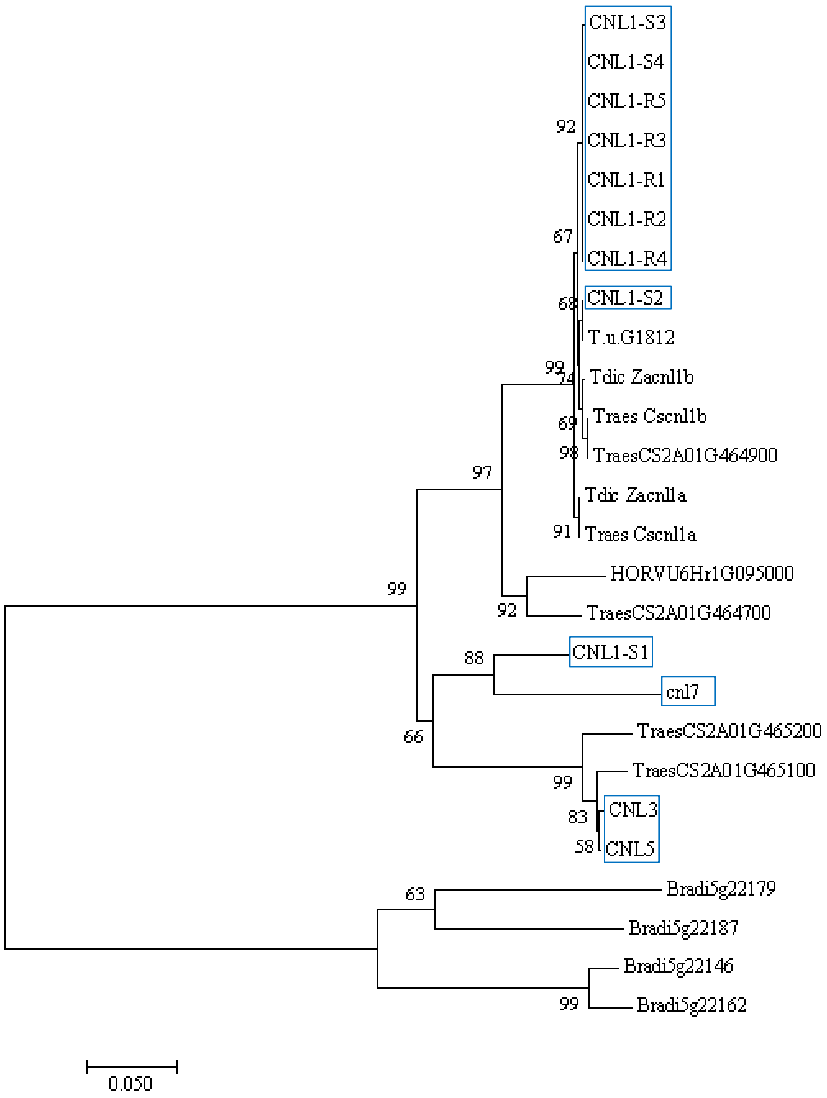

S9 Fig. Phylogenetic tree of NLR genes related to *CNL1*. Neighbor-Joining tree analysis including *CNL1* haplotypes, linked *CNL3*, *CNL5* and *cnl7* genes (blue squares), and the closest predicted genes from *T. urartu* (T.u.), *T. dicoccoides* (Tdic), *Triticum aestivum* (Traes), *Hordeum vulgare* (HORVU), and *Brachypodium distachyon* (Bradi). Coding DNA sequences were aligned with muscle as implemented in Mega 7, and phylogenetic trees were then generated using the pair-wise deletion method (bootstrap confidence values based on 1000 iterations).
