# Supplementary material for: Identification and characterization of wheat stem rust resistance gene Sr21 effective against the Ug99 race group at high temperature
Source: PLoS Genet. 2018 Apr 3;14(4):e1007287. doi: 10.1371/journal.pgen.1007287 (PMC5882135; doi:10.1371/journal.pgen.1007287)
Supplement: S10 Fig — The undigested 951-bp PCR products (black arrow) are present in genotypes DV92 (R1), G3116 (R3), PI 306540 (R2), CSSr21 (R1), PI 427971-R (R4) and PI 427796 (R5) carrying the different Sr21 resistant haplotypes. A digested band of 836-bp (yellow arrowhead, 115 bp band out of the gel) was detected in T. monococcum genotypes PI 427971-S and PI 538540 that carry the Sr21 susceptible haplotypes S3 or S4. No amplification product was detected with these primers for susceptible genotypes of T. monococcum PI 272557 (S1), T. urartu PI 428227 (S2), PI 428183 (S2), and the related susceptible haplotypes present in Kronos, Fielder and Chinese Spring (CS) (S8 Fig and S8 Table). (PDF) [file pgen.1007287.s010.pdf]

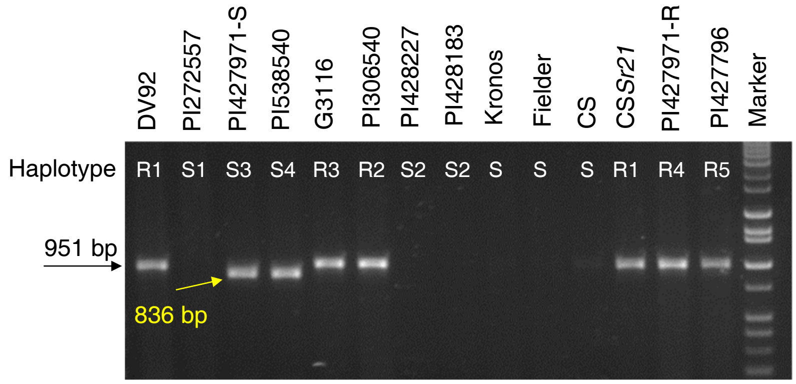

S10 Fig. *Sr21* diagnostic PCR marker Sr21TRYF5R5 digested with *Nsi*I. The undigested 951-bp PCR products (black arrow) are present in genotypes DV92 (R1), G3116 (R3), PI 306540 (R2), CS*Sr21* (R1), PI 427971-R (R4) and PI 427796 (R5) carrying the different *Sr21* resistant haplotypes. A digested band of 836-bp (yellow arrowhead, 115 bp band out of the gel) was detected in *T. monococcum* genotypes PI 427971-S and PI 538540 that carry the *Sr21* susceptible haplotypes S3 or S4. No amplification product was detected with these primers for susceptible genotypes of *T. monococcum* PI 272557 (S1), *T. urartu* PI 428227 (S2), PI 428183 (S2), and the related susceptible haplotypes present in Kronos, Fielder and Chinese Spring (CS) (S8 Fig. and S8 Table).
