# Supplementary material for: Identification and characterization of wheat stem rust resistance gene Sr21 effective against the Ug99 race group at high temperature
Source: PLoS Genet. 2018 Apr 3;14(4):e1007287. doi: 10.1371/journal.pgen.1007287 (PMC5882135; doi:10.1371/journal.pgen.1007287)
Supplement: S1 Table — Primers for high density genetic map, screening of the T. monococcum BAC library, haplotype analysis, mutant screening, marker-assisted selection (MAS), expression analysis, 3’ and 5’ RACE, cloning and screening for transgenic studies, and copy number assays. (PDF) [file pgen.1007287.s011.pdf]

Supplementary Tables

S1 Table. Primers used in this study. Primers for high density genetic map, screening of the *T. monococcum* BAC library, haplotype analysis, mutant screening, marker-assisted selection (MAS), expression analysis, 3’ and 5’ RACE, cloning and screening for transgenic studies, and copy number assays.

Marker	Marker (enzyme)	Primer sequence 5'-3' (Forward)	Primer sequence 5'-3' (Reverse)	Function
FD527726	Indel	CGGCATCAATAGGAGAAGA	TAGGATACGTGACCCAGGA	Fine map
EX594406	CAP (*Xmn*I)	TCAACAACTTCAACAAGGC	AACAAGAGAACGAGCATCG	Fine map
CD887748	Indel	CTCTCATTGTTTCTAGGGGATT	TAGGATTTCAGCAGCATTTG	Fine map
CJ961291	CAP (*Sty*I)	CCCAAGAGATGAATCAAACC	CAACTGAAGCTGCTCAAAAG	Fine map
Cscnl20	Dominant	TACTTCTAACATTCCCCCTC	ATGAAACTTTGGTACCGTCT	Fine map
Cscnl21	Dominant	GCCTGGATGTGGTTCAAGAC	AGATGAGAGCACCCGTTTTC	Fine map
Cscnl22	Dominant	AAATGCACAGCATTTACGC	TCACTAGATGATCCCCCCA	Fine map
Cscnl23	Dominant	GGGGCAGGACTAAGAAACA	TCCAAATGAGGGCAATAACT	Fine map
205J7TF2-1R2		GATCCATGCCACACTAAACAATG	TGACCCCGAATCCGAAAA	Tm BAC lib.
590TF3R3		GATCGTGTTGTATGTGCCAG	GCTCTTTAGGTTAAGGGGGT	Tm BAC lib.
80L18F1R1		GACATATCGTCGTCTTGCTCC	CAGGACTCCCTCGGCGTCACTT	Tm BAC lib.
655G5F1R1		TGAATAAGAGTAGCGACAGAT	GATAAGAGCAAGACAAGGAC	Tm BAC lib.
557I2F14R14		AATCTTTATGCACGTGTCGG	TTAGCTCAGCTTGCTTCCTCT	Tm BAC lib.
179C18F7R7		GGAATACGGACGGAGAGGGA	CAGGCACGGAAACAGGACAG	Tm BAC lib.
S21CNL1F1R1		CCTACTGCTTGTACCATTTGTTCAG	GTTATTTGGCTTGTGGCGCTG	Haplotype
S21CNL1F3R3		TGCAGCGCCACAAGCCAAATA	TGTAACCCCCACAGAACTCCAGAT	Haplotype
S21CNL1F8R8		TCAACTGCTTTCACATTCGC	CCTTATGCTTTCCTGCTTCC	Haplotype
S21CNL1F5R5		TGTCACACTTAGCAACCGT	GGTACTAGCAACAACCAGAA	Mutants screening
S21CNL1F10R10		ATCAGCTCTATATACAATTCGT	GCATTTTAGTCAACACCAAC	Mutants screening
S21CNL1F12R24		GGTTGCGCATACTTATCAGAAAT	TCCATCCACCCCTGTCCTT	Mutants screening
S21CNL1F17R14		TAATAGTGAATCTTCTATGCAGTAG	AGGATGGTGCTCGCGGTTG	Mutants screening
S21CNL1F26R26		TGTCCAATCCCATCTTCTGTCT	GCCCTTATGCTTTCCTGCT	Mutants screening
S21CNL1F22R20		CTTCATCTGGAGGAGTGTAGGGAG	AGGGACGGAGGAAGCAGC	Mutants screening
Sr21TRYF5R5	CAP (*Nsi*I)	CCTAGAGAAACGGAAGGGACCA	TGTGAGCTGTTTGCAGAAGTGTG	MAS
Sr21qRTF1R1		CAGAAGGGAAAGGTTGTAGTGCTC	TCAGTTATTTGGCTTGTGGCG	Expression analysis
SR215’OUT-R3			CACGAGTCCAGGCTTCCATACG	5’ RACE
SR215’IN-R4			CCAAGCAACCCCGACATCTCTC	5’ RACE
SR213’OUT-F1		CCAAGAGCTTCAATTTCGGAGAT		3’ RACE
SR213’IN-F2		GAAGCTAAGGGTGAGGATTGATC		3’ RACE
SR21TransF1-2R1		CACCGTCGACGCACAAGGAACCAGAGGAGGA	TCCTATCTCAAAACTGCCAACA	Transgenic
SR21TransF2R2		CACCACTGGATGTGGGCATTTCGGGC	AGGACCTAGGGGGTGGGGGTTCTCGGTTGA	Transgenic
M13-pEntry-F/R		GTAAAACGACGGCCAG	CAGGAAACAGCTATGAC	Transgenic
Hptmiki-F/R		GGCCTCCAGAAGAAGATGTTGG	GAGCCTGACCTATTGCATCTCC	Transgenic
SR21CopyNumF1R1		CTGTGACCTTGTCTGGACTGC	CTCAGAGCCGAACTGTAGGAGT	Copy number
SR21-Probe		FAM-TGACGGATCTGTCCTTTCCACACAC-BHQ1		Copy number
CO2F1/R1		TGCTAACCGTGTGGCATCAC	GGTACATAGTGCTGCTGCATCTG	Copy number
CO2-Probe		CY5-CATGAGCGTGTGCGTGTCTGCG-BHQ3		Copy number
