# Supplementary material for: Identification and characterization of wheat stem rust resistance gene Sr21 effective against the Ug99 race group at high temperature
Source: PLoS Genet. 2018 Apr 3;14(4):e1007287. doi: 10.1371/journal.pgen.1007287 (PMC5882135; doi:10.1371/journal.pgen.1007287)
Supplement: S2 Table — Number of insertions in each transgenic event based on T1 plants and a TaqMan copy number assay of the transgenic plants relative to CSSr21 (1 copy). (PDF) [file pgen.1007287.s012.pdf]

S2 Table. **Estimated copy number of transgenic insertions.** Number of insertions in each transgenic event based on T_1 plants and a TaqMan copy number assay of the transgenic plants relative to CS*Sr21* (1 copy).

		Transgenic family				
		T_1Sr21-02	T_1Sr21-03	T_1Sr21-04	T_1Sr21-09	T_1Sr21-14
CNL1 DNA marker [1]	present	49	57	56	47	44
	absent	0	0	0	1	1
χ^2 *P* value	1 copy	<0.0001	<0.0001	<0.0001	0.0002	0.0004
	2 copies	0.0707	0.0513	0.0533	0.233	0.2643
	3 copies	0.3778	0.3415	0.3458	**0.7711**	**0.7212**
	4 copies	**0.6611**	0.6364	0.6393	0.0601	0.0489
	5 copies	**0.8268**	**0.8134**	0.815	<0.0001	<0.0001
	6 copies	0.9129	0.9061	**0.9069**	<0.0001	<0.0001
TaqMan	Transgene[2] /CS*Sr21*	4.57	4.98	6.02	3.03	3.71
Copy No.	from genotype	4 to 5	5	6	3	3
	from phenotype	2	2	2	2	1
Expression[3]	T_1/ CS*Sr21*	2.55	4.77	5.72	4.33	2.97

[1] Genotyped with markers S21CNL1F5R5 (digested with *Sph*I) and Sr21TRYF6-1R5 (Table S1). The estimated number integrates information from TaqMan and segregation data.

[2] Since T_1s were segregating for copy number, we divided the average of the two highest copy number values in the progeny by CS*Sr21* average copy number.

[3] We divided the average of the two highest expression values in the progeny by CS*Sr21* expression value.
