# Supplementary material for: Identification and characterization of wheat stem rust resistance gene Sr21 effective against the Ug99 race group at high temperature
Source: PLoS Genet. 2018 Apr 3;14(4):e1007287. doi: 10.1371/journal.pgen.1007287 (PMC5882135; doi:10.1371/journal.pgen.1007287)
Supplement: S3 Table — Alternative splicing forms were identified from multiple 3' RACE reactions (total RNAs of G3116 from 24°C / Inoculated / 6 d, 24°C / Mock-inoculated / 6 d, 16°C / Inoculated / 6 d and 16°C / Mock-inoculated / 6 d). Eighty colonies (using TA-cloning) from every 3' RACE reaction were PCR amplified and sequenced using the Sanger method. Eighty clones were analyzed for each of the four conditions. For the χ2 analysis, the five less frequent alternative splice forms were merged (CNL1-3, CNL1-4, CNL1-7, CNL1-8, CNL1-9, and CNL1-10). (PDF) [file pgen.1007287.s013.pdf]

S3 Table. Alternative splicing forms. Alternative splicing forms were identified from multiple 3' RACE reactions (total RNAs of G3116 from 24 °C / Inoculated / 6 d, 24 °C / Mock-inoculated / 6 d, 16 °C / Inoculated / 6 d and 16 °C / Mock-inoculated / 6 d). Eighty colonies (using TA-cloning) from every 3' RACE reaction were PCR amplified and sequenced using the Sanger method. Eighty clones were analyzed for each of the four conditions. For the χ^2 analysis, the five less frequent alternative splice forms were merged (*CNL1*-3, *CNL1*-4, *CNL1*-7, *CNL1*-8, *CNL1*-9, and *CNL1*-10).

	24 °C		16 °C	
	Inoculated	Mock-inoculated	Inoculated	Mock-inoculated
CNL1-1	55%	61.20%	42.50%	46.30%
CNL1-2	10%	8.80%	35.00%	37.50%
CNL1-3	2.50%	2.50%	0%	2.50%
CNL1-4	7.50%	3.80%	2.50%	2.50%
CNL1-5	7.50%	16.20%	7.50%	2.50%
CNL1-6	10%	5.00%	3.70%	2.50%
CNL1-7	2.50%	2.50%	7.50%	0%
CNL1-8	2.50%	0%	1.30%	1.20%
CNL1-9	2.50%	0%	0%	1.30%
CNL1-10	0%	0%	0%	3.80%

χ^2 inoculation P = 0.81, χ^2 temperature P = 0.002
