# Supplementary material for: Identification and characterization of wheat stem rust resistance gene Sr21 effective against the Ug99 race group at high temperature
Source: PLoS Genet. 2018 Apr 3;14(4):e1007287. doi: 10.1371/journal.pgen.1007287 (PMC5882135; doi:10.1371/journal.pgen.1007287)
Supplement: S4 Table — Transcript levels at two temperatures (16°C and 24°C), two Pgt treatments (inoculated and mock-inoculated), and four collection time points after inoculation (0 hour, 1 dpi, 3 dpi and 6 dpi). (A) Three-way ANOVA at time 0 immediately after inoculation and transfer from greenhouse at 20°C to the chambers at 16°C and 24°C. (B) Four-way ANOVA at 1, 3 and 6 dpi. (PDF) [file pgen.1007287.s014.pdf]

**S4 Table.** ***CNL1*** **expression levels.** Transcript levels at two temperatures (16 °C and 24 °C), two *Pgt* treatments (inoculated and mock-inoculated), and four collection time points after inoculation (0 hour, 1 dpi, 3 dpi and 6 dpi). **(A)** Three-way ANOVA at time 0 immediately after inoculation and transfer from greenhouse at 20 °C to the chambers at 16 °C and 24 °C. **(B)** Four-way ANOVA at 1, 3 and 6 dpi.

**A** 0 hour

| Source | DF | Type III SS | Mean Square | F Value | Pr > F |
|---|---|---|---|---|---|
| Genotype | 1 | 1.28807 | 1.28807 | 8.88 | **0.0089** |
| Temp | 1 | 0.02940 | 0.02940 | 0.20 | 0.6587 |
| Genotype*Temp | 1 | 0.00001 | 0.00001 | 0.00 | 0.9924 |
| Inoc | 1 | 0.04806 | 0.04806 | 0.33 | 0.5730 |
| Genotype*Inoc | 1 | 0.18166 | 0.18166 | 1.25 | 0.2797 |
| Temp*Inoc | 1 | 0.00180 | 0.00180 | 0.01 | 0.9126 |
| Genotype*Temp*Inoc | 1 | 0.00510 | 0.00510 | 0.04 | 0.8536 |

**B** 1, 3 and 6 days

| Source | DF | Type III SS | Mean Square | F Value | Pr > F |
|---|---|---|---|---|---|
| Genotype | 1 | 0.01434 | 0.01434 | 0.15 | 0.7046 |
| Day | 2 | 0.90101 | 0.45051 | 4.56 | **0.0145** |
| Genotype*Day | 2 | 0.42759 | 0.21379 | 2.17 | 0.1241 |
| Temp | 1 | 17.44829 | 17.44829 | 176.71 | **<.0001** |
| Genotype*Temp | 1 | 0.53423 | 0.53423 | 5.41 | 0.0236 |
| Day*Temp | 2 | 2.19264 | 1.09632 | 11.10 | **<.0001** |
| Inoc | 1 | 30.95796 | 30.95796 | 313.52 | **<.0001** |
| Genotype*Inoc | 1 | 0.03529 | 0.03529 | 0.36 | 0.5523 |
| Day*Inoc | 2 | 3.13951 | 1.56976 | 15.90 | **<.0001** |
| Temp*Inoc | 1 | 0.66394 | 0.66394 | 6.72 | **0.0121** |
