# Supplementary material for: Identification and characterization of wheat stem rust resistance gene Sr21 effective against the Ug99 race group at high temperature
Source: PLoS Genet. 2018 Apr 3;14(4):e1007287. doi: 10.1371/journal.pgen.1007287 (PMC5882135; doi:10.1371/journal.pgen.1007287)
Supplement: S5 Table — P values of transcript levels of PR genes in CSSr21 and G3116 plants 6 dpi with Pgt race BCCBC or mock-inoculated at high (24°C) and low (16°C) temperatures. CSSr21 and G3116 indicate P values for the individual two-way ANOVAS. The last row indicates the ratio between the diploid and hexaploid average expression of the PR gene in the Pgt inoculated plants at 24°C. (PDF) [file pgen.1007287.s015.pdf]

S5 Table. Three-way ANOVA. *P* values of transcript levels of *PR* genes in CS*Sr21* and G3116 plants 6 dpi with *Pgt* race BCCBC or mock-inoculated at high (24 °C) and low (16 °C) temperatures. CS*Sr21* and G3116 indicate *P* values for the individual two-way ANOVAS. The last row indicates the ratio between the diploid and hexaploid average expression of the *PR* gene in the *Pgt* inoculated plants at 24 °C.

Variety	Source	PR1	PR2	PR3	PR4	PR5	PR9
Combined	Genotype	<0.0001	0.0012	<0.0001	<0.0001	<0.0001	<0.0001
	Temperature	0.1215	0.0138	0.0081	0.0653	<0.0001	0.3403
	Inoculation	<0.0001	<0.0001	<0.0001	<0.0001	<0.0001	<0.0001
	Inoculation*Temp.	<0.0001	<0.0001	<0.0001	<0.0001	<0.0001	<0.0001
	Genotype*Temp.	0.7018	0.4017	0.6498	0.9389	0.0002	0.0164
	Genotype*Inoc.	0.0220	<0.0001	0.2735	0.3623	0.0001	0.0009
CS*Sr21*	Inoculation	0.0004	<0.0001	0.0009	<0.0001	<0.0001	0.0017
	Temp.	0.3522	0.0362	0.0975	0.2816	0.0002	0.3005
	Inoculation*Temp.	0.0032	<0.0001	0.0015	0.0004	<0.0001	0.0067
G3116	Inoculation	<0.0001	<0.0001	<0.0001	<0.0001	<0.0001	<0.0001
	Temp.	0.1937	0.2007	0.0443	0.1116	0.1343	0.0187
	Inoculation*Temp.	0.0145	0.0026	0.0001	<0.0001	0.0008	0.0012
2x / 6x	High T, inoculated	5.5	1.5	7.1	3.4	4.6	14.6
