# Supplementary material for: Identification and characterization of wheat stem rust resistance gene Sr21 effective against the Ug99 race group at high temperature
Source: PLoS Genet. 2018 Apr 3;14(4):e1007287. doi: 10.1371/journal.pgen.1007287 (PMC5882135; doi:10.1371/journal.pgen.1007287)
Supplement: S6 Table — Sporulation size was determined at 14 dpi, whereas Pgt/host DNA ratio and area of fungal growth detected by fluorescence were determined at 5 dpi. Experiments with BCCBC were performed at 16°C and 24°C and those for TTKSK were performed at 16°C and 20°C and were reported before [20]. Six replications were used per treatment combination, with the exception of sporulation size for race TTKSK where only three replications were used. Normality of residuals of the transformed data was confirmed using Shapiro-Wilk test. (PDF) [file pgen.1007287.s016.pdf]

**S6 Table. Pathogen growth at different temperatures in susceptible and *Sr21*-resistant diploid and hexaploid wheat.** Sporulation size was determined at 14 dpi, whereas *Pgt*/host DNA ratio and area of fungal growth detected by fluorescence were determined at 5 dpi. Experiments with BCCBC were performed at 16 °C and 24 °C, and those for TTKSK were performed at 16 °C and 20 °C and were reported before [20]. Six replications were used per treatment combination, with the exception of sporulation size for race TTKSK where only three replications were used. Normality of residuals of the transformed data was confirmed using Shapiro-Wilk test.

| | Avg. sporulation area | | *Pgt*/wheat DNA | Fluorescence |
|---|---|---|---|---|
| | BCCBC | TTKSK | BCCBC | BCCBC |
| Temperature | 0.5940 | <0.0001 | <0.0001 | <0.0001 |
| Ploidy | <0.0001 | <0.0001 | 0.0080 | 0.0010 |
| *Sr21* | <0.0001 | <0.0001 | <0.0001 | <0.0001 |
| Temp x Ploidy | 0.0121 | <0.0001 | 0.2493 | 0.0227 |
| Temp x *Sr21* | <0.0001 | <0.0001 | <0.0001 | <0.0001 |
| Ploidy x *Sr21* | <0.0001 | 0.2337 | 0.0382 | 0.0963 |
| Temp x Ploidy x *Sr21* | 0.5207 | 0.2420 | 0.0018 | 0.6712 |
| Replications | 6 | 3 | 6 | 6 |
| Normality of residuals | 0.6912 | 0.2696 | 0.5069 | 0.5339 |
