# Supplementary material for: Identification and characterization of wheat stem rust resistance gene Sr21 effective against the Ug99 race group at high temperature
Source: PLoS Genet. 2018 Apr 3;14(4):e1007287. doi: 10.1371/journal.pgen.1007287 (PMC5882135; doi:10.1371/journal.pgen.1007287)
Supplement: S7 Table — Infection types in diploid accessions (Triticum monococcum subsp. aegilopoides, Triticum monococcum subsp. monococcum and Triticum urartu) to five Pgt races TRTTF, TTKSK, TTTTF, QFCSC, and MCCFC. Infection types shown here were based on the previous study [49]. (PDF) [file pgen.1007287.s017.pdf]

S7 Table. Infection types. Infection types in diploid accessions (*Triticum monococcum* subsp. *aegilopoides*, *Triticum monococcum* subsp. *monococcum* and *Triticum urartu*) to five *Pgt* races TRTTF, TTKSK, TTTTF, QFCSC, and MCCFC. Infection types shown here were based on the previous study [49]

Accession [a]	Species	TRTTF	TTKSK	TTTTF	QFCSC	MCCFC	Gene Postulation
PI 245726, PI 272556, PI 306527, PI 306532, PI 352274, PI 401411, PI 427461, PI 427562, PI 427835, PI 427937, PI 427971, PI 538540, PI 538546, PI 538552	*T. monococcum* subsp. *aegilopoides*	3 to 4	3 to 4	3 to 4	3 to 4	3 to 4	no *Sr21*
PI 272557, PI 119422, PI 167526, PI 221416, PI 277138, PI 277140, PI 306542, PI 352475, PI 355526, PI 355528, PI 542473	*T. monococcum* subsp. *monococcum*	3 to 4	3 to 4	3 to 4	4	3+ to 4	no *Sr21*
PI 352270, PI 355453, PI 427452, PI 427484, PI 427497, PI 427527, PI 427545, PI 427555, PI 427580, PI 427592, PI 427603, PI 427688, PI 427693, PI 427796, PI 427808, PI 427992	*T. monococcum* subsp. *aegilopoides*	3+ to 4	1/2/12Z/22+	3 to 4	3 to 4	1;/1	*Sr21*
CItr 13963, CItr 14520, CItr 17655, CItr 17657, PI 94743, PI 168806, PI 190942, PI 221393, PI 225164, PI 237659, PI 266844, PI 277130, PI 277135, PI 286068, PI 295058, PI 306547, PI 307984, PI 345242, PI 352473, PI 352484, PI 355538, PI 355547, PI 355548, PI 362610, PI 377668, PI 418583	*T. monococcum* subsp. *monococcum*	3 to 4	;/1/2/2+/22+	3 to 4	3 to 4	0;/;1	*Sr21*
PI 428227, PI 428183, PI 428210, PI 428231, PI 428211, PI 428195, PI 538728, PI 503319, PI 428235, PI 538726, PI 428193, PI 428213, PI 428217, PI 428197, PI 428212, PI 428216, PI 428196, PI 428199, PI 428186, PI 428203, PI 428238, PI 428180, PI 428181, PI 428182, CItr 17664, CItr 17666, PI 428327, PI 428328, PI 487265, PI 487266, PI 487267, PI 487268, PI 487269, PI 487270, PI 487271, PI 487272, PI 428239, PI 428232, PI 428233, CItr 1766	*Triticum urartu*	4	2- or 4	3+ to 4	3 to 4	3 to 4	no *Sr21*

[a] *T. monococcum* lines DV92 and PI 306540 were not included in this table since they contain additional Ug99 resistance genes.
