# Supplementary material for: Identification and characterization of wheat stem rust resistance gene Sr21 effective against the Ug99 race group at high temperature
Source: PLoS Genet. 2018 Apr 3;14(4):e1007287. doi: 10.1371/journal.pgen.1007287 (PMC5882135; doi:10.1371/journal.pgen.1007287)
Supplement: S8 Table — CNL1 haplotypes among a collection of 74 T. monococcum and 40 T. urartu accessions. (PDF) [file pgen.1007287.s018.pdf]

**S8 Table. Haplotypes of *CNL1*.** *CNL1* haplotypes among a collection of 74 *T. monococcum* and 40 *T. urartu* accessions.

| Haplotype | Acc. No. | Accessions |
|---|---|---|
| R1 | 28 | ***T. monococcum***: DV92, PI 119422-R[a], PI 94743, PI 237659, PI 266844, PI 286068, PI 306547, PI 352484, CItr 13963, CItr 14520, CItr 17657, PI 190942, PI 221393, PI 277135, PI 307984, PI 345242, PI 352473, PI 355547, PI 355548, PI 418583, PI 225164-R[a], PI 427580, PI 427603, PI 427592, PI 427497, PI 427452, PI 355453, PI 352270 |
| R2 | 6 | PI 306540, PI 10474 (CItr2433), CItr 17655, PI 168806, PI 277130, PI 295058 |
| R3 | 1 | G3116 (=PI 427992) |
| R4 | 6 | PI 427971-R[a], PI 427808, PI 427688, PI 427555, PI 427484, PI 427527 |
| R5 | 3 | PI 427796, PI 427693, PI 427545 |
| S1 | 25 | PI 272557, PI 272556, PI 306527, PI 306532, PI 352274, PI 427461, PI 427835, PI 427937, PI 538546, PI 538552, PI 167526, PI 221416, PI 277138, PI 277140, PI 306542, PI 352475, PI 355526, PI 355528, PI 542473, PI 355538[b], PI 362610[b], PI 377668[b], PI 355523, PI 119422-S[a], PI 225164-S[a] |
| S2 | 43 | ***T. urartu*** (but misclassified as *T. monococcum*): PI 245726[c], PI 401411[c], PI 427562[c]. ***T. urartu***: PI 428227, PI 428183, PI 428210, PI 428231, PI 428211, PI 428195, PI 538728, PI 503319, PI 428235, PI 538726, PI 428193, PI 428213, PI 428217, PI 428197, PI 428212, PI 428216, PI 428196, PI 428199, PI 428186, PI 428203, PI 428238, PI 428180, PI 428181, PI 428182, CItr 17664, CItr 17666, PI 428327, PI 428328, PI 487265, PI 487266, PI 487267, PI 487268, PI 487269, PI 487270, PI 487271, PI 487272, PI 428239, PI 428232, PI 428233, CItr 1766. |
| S3 | 1 | ***T. monococcum***: PI 427971-S[a] |
| S4 | 1 | ***T. monococcum*:** PI 538540 |

[a] Heterozygous accessions showing resistant (-R) and susceptible (-S) individuals.

[b] Three accessions with the susceptible haplotype (S1) showed resistance to MCCFC and susceptibility to BCCBC suggesting that they carry a *Pgt* resistance gene different from *Sr21*.

[c] Three accessions classified by the NSGC as *Triticum monococcum* subsp. *aegilopoides* carry similar alleles to *T. urartu* for *CNL1* and for other 12 genes and are likely misclassified.
